# An Automated Segmentation Pipeline for Intratumoural Regions in Animal Xenografts Using Machine Learning and Saturation Transfer MRI

**DOI:** 10.1038/s41598-020-64912-6

**Published:** 2020-05-15

**Authors:** Wilfred W. Lam, Wendy Oakden, Elham Karami, Margaret M. Koletar, Leedan Murray, Stanley K. Liu, Ali Sadeghi-Naini, Greg J. Stanisz

**Affiliations:** 1 Physical Sciences, Sunnybrook Research Institute, Toronto, ON Canada; 2 Medical Biophysics, University of Toronto, Toronto, ON Canada; 3 Electrical Engineering and Computer Science, Lassonde School of Engineering, York University, Toronto, ON Canada; 4 Radiation Oncology, University of Toronto, Toronto, ON Canada; 5 Biological Sciences, Sunnybrook Research Institute, Toronto, ON Canada; 6 Radiation Oncology, Sunnybrook Health Sciences Centre, Toronto, ON Canada; 7 Neurosurgery and Paediatric Neurosurgery, Medical University of Lublin, Lublin, Poland

**Keywords:** Tumour heterogeneity, Machine learning, Cancer imaging

## Abstract

Saturation transfer MRI can be useful in the characterization of different tumour types. It is sensitive to tumour metabolism, microstructure, and microenvironment. This study aimed to use saturation transfer to differentiate between intratumoural regions, demarcate tumour boundaries, and reduce data acquisition times by identifying the imaging scheme with the most impact on segmentation accuracy. Saturation transfer-weighted images were acquired over a wide range of saturation amplitudes and frequency offsets along with T_1_ and T_2_ maps for 34 tumour xenografts in mice. Independent component analysis and Gaussian mixture modelling were used to segment the images and identify intratumoural regions. Comparison between the segmented regions and histopathology indicated five distinct clusters: three corresponding to intratumoural regions (active tumour, necrosis/apoptosis, and blood/edema) and two extratumoural (muscle and a mix of muscle and connective tissue). The fraction of tumour voxels segmented as necrosis/apoptosis quantitatively matched those calculated from TUNEL histopathological assays. An optimal protocol was identified providing reasonable qualitative agreement between MRI and histopathology and consisting of T_1_ and T_2_ maps and 22 magnetization transfer (MT)-weighted images. A three-image subset was identified that resulted in a greater than 90% match in positive and negative predictive value of tumour voxels compared to those found using the entire 24-image dataset. The proposed algorithm can potentially be used to develop a robust intratumoural segmentation method.

## Introduction

Tumours are highly heterogeneous. Not only do they vary considerably between different individuals, but a single tumour often demonstrates regional variations in cell density, cell death, vasculature, and metabolic activity, among other factors^1^. These subregions can be due to genetic or local microenvironmental differences^1,2^. Differentiation between active tumour and necrosis is of particular clinical interest^3,4^ since heterogeneity is often predictive of survival, therapeutic response, or metastatic potential^2,5,6^.

There is a diagnostic advantage to the segmentation of heterogeneous tumours prior to further analysis. Considering the tumour as a single entity and calculating whole-tumour metrics, such as perfusion parameters, can result in a loss of correlation between biomarkers^5^. Magnetic resonance imaging (MRI) is ideal for identifying intratumoural regions as it is non-invasive and does not utilize ionizing radiation. While tumour heterogeneity can be observed on conventional T_2_-weighted and post-contrast agent injection T_1_-weighted^7,8^ MRI, quantitative techniques are generally required in order to accurately segment intratumoural regions^5,9^. Manual segmentation is certainly possible^2,10^. However, it is time consuming, subjective, and typically based on a single image contrast. It is also exacerbated by the fact that tumour boundaries are often irregular and intratumoural regions may not be contiguous.

Automatic segmentation can be performed by fitting a model to the imaging data and thresholding the model parameters^11–13^. It can also be done using machine learning. Specific methods include the use of convolutional neural networks^7,8^, Gaussian mixture modelling^14–17^, k-means clustering^9,17–19^, non-negative matrix factorization^20,21^, and other techniques^17,22^. Quantitative MRI data used by these automatic segmentation routines include proton density (M_0_)^9,18^, transverse relaxation time (T_2_)^9,14,17,18,22^, effective transverse relaxation time (T_2_*)^8,12,14,17^, diffusion-weighted imaging (DWI) model parameters^8,9,11,14,15,17,18,21,22^, and dynamic contrast enhancement (DCE) model parameter maps^8,12,14,16,20^. While these methods are promising, these are not the only options for quantitative imaging.

Saturation transfer MRI is sensitive to differences in tumour metabolism, which differ between intratumoural regions^1^, and can also be particularly useful in the characterization of different tumour types^23^, since it does offer superb tissue contrast, in comparison to other methods, without a need for exogenous contrast agents. The saturation transfer MRI contrast mechanism reflects the exchange rate of magnetization between hydrogen nuclei in water and other molecular pools that include macromolecules and dissolved proteins, as well relative pool sizes and their intrinsic magnetic resonance properties such as the longitudinal and transverse relaxation times (T_1_ and T_2_, respectively) of each pool. In saturation transfer-prepared pulse sequences, magnetization is reduced by an RF (radiofrequency) saturation pulse across a range of frequencies corresponding to the exchanging molecules. When high saturation amplitudes and large frequency offsets relative to the water resonance are used, these sequences are typically referred to as magnetization transfer (MT) and are sensitive to the exchange of hydrogen nuclei in semisolid macromolecules (mostly lipid bilayers)^24–26^ with those of water. At lower saturation amplitudes and smaller frequency offsets, they are mostly sensitive to the exchange in chemical groups in dissolved proteins (e.g., amide^27^, amine^28^, guanidinium^29,30^, and hydroxyl^28^) and the mechanism is termed Chemical Exchange Saturation Transfer (CEST) or relayed-Nuclear Overhauser Effect (NOE). Importantly, CEST, unlike DCE, does not require the injection of an exogenous contrast agent, which can be an issue for renally compromised patients^31^ and a complication for longitudinal preclinical studies. CEST MRI in oncology is an active area of research^32,33^. In a previous segmentation study involving saturation transfer, Zhang *et al*. combined manual tumour delineation with MT and contrast to segment tumour from necrosis, which has much lower MT and relayed-NOE contrast^10^.

The goal of the present study was to develop an automated algorithm to segment intratumoural regions as well as the surrounding tissue in a xenograft model of prostate cancer using only saturation transfer MRI data and T_1_ and T_2_ maps. This would allow a secondary use of this data, which originally was intended for studying metabolism in tumours^23^. The algorithm was validated using histopathology and was tested for robustness using leave-one-out cross-validation. The trade-off between the number of image contrast types and segmentation accuracy was investigated for the purpose of minimizing data acquisition and the images with the largest impact on accuracy were found via feature selection. Finally, the MT and CEST effects in active tumour vs. necrosis/apoptosis were also quantitatively compared.

## Results

### Description of tumours

**Figure 1:**
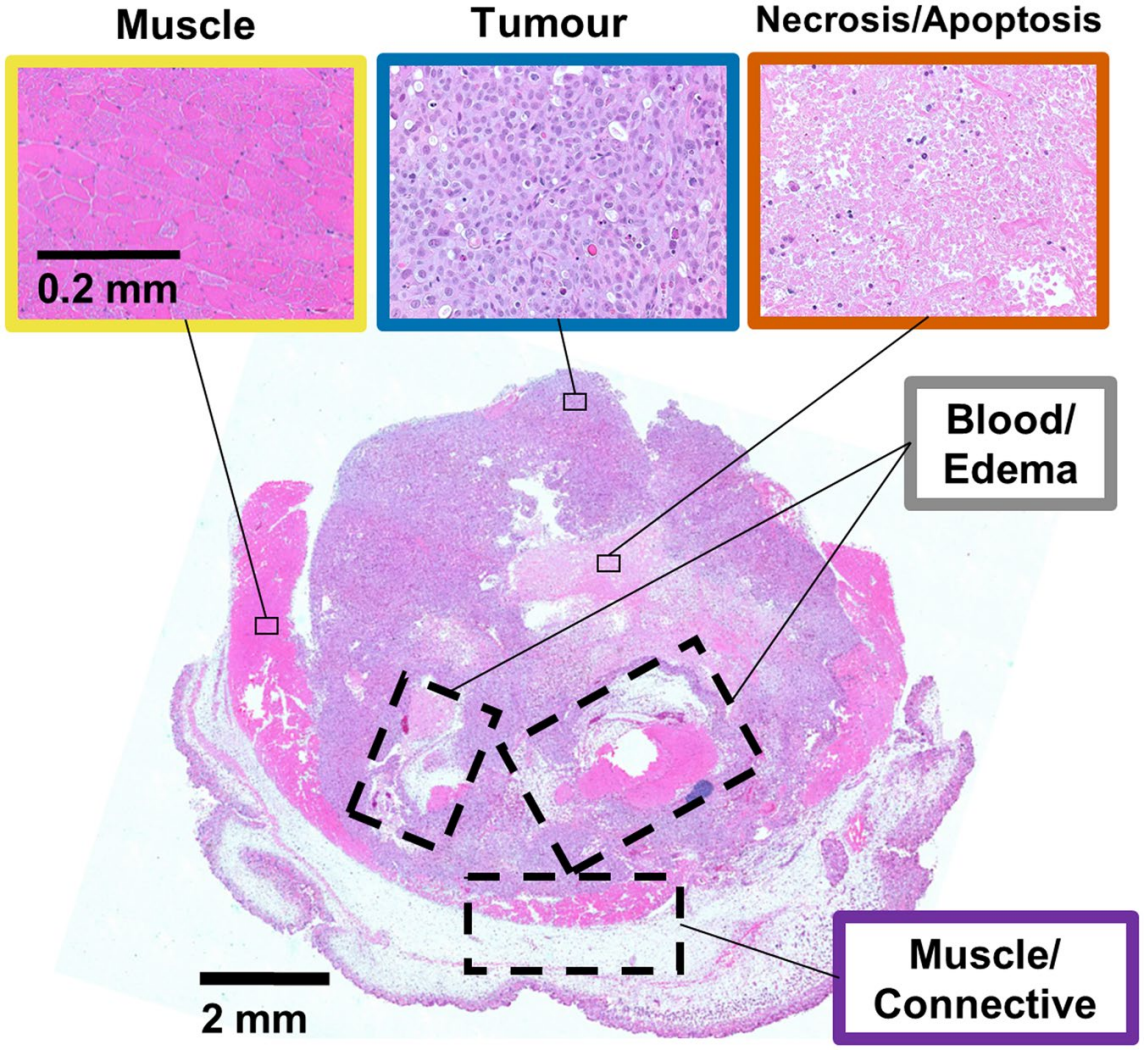
H&E stained section with details of three clusters for an illustrative tumour. Images are presented at 5× and 20× magnification for the whole-tissue slice and details, respectively.

Of the 34 DU145 human prostate adenocarcinoma tumour xenografts included in this study, 33 ranged in size from 33 to 810 mm^3^ (with a mean ± SD of 259 ± 210 mm^3^) and one (shown in Fig. 1) was ~1,500 mm^3^ with a particularly large region of edema. Imaging and histology were acquired 46 ± 12 days post injection of tumour cells.

### Histopathology

Most tumours were heterogeneous and comprised of a complex mixture of muscle cells, tumour cells, necrotic and apoptotic cells, blood cells, and regions of inter-cellular edema. Figure 1 shows an example of a particularly complex tumour. Most of the 34 tumours were largely active, indicated by colourless terminal deoxynucleotidyl transferase dUTP nick end labelling (TUNEL) images (Supplementary Fig. S1). Eight had significant edema indicated by the large hyperintense regions in the T_2_-weighted images. Edema presented a challenge for interpretation of histopathology since the voxel size and liquid signal often masked an intricate microenvironment of muscle cells, leukocytes, fibroblasts, tumour cells, and fibrotic necrosis, as illustrated in the regions of blood/edema in the haematoxylin and eosin (H&E) histology (boxes with dashed borders in Fig. 1). Twelve tumours had large areas of necrosis/apoptosis indicated by the significant brown staining in the TUNEL images (Supplementary Fig. S1).

### Optimization of segmentation pipeline

**Figure 2:**
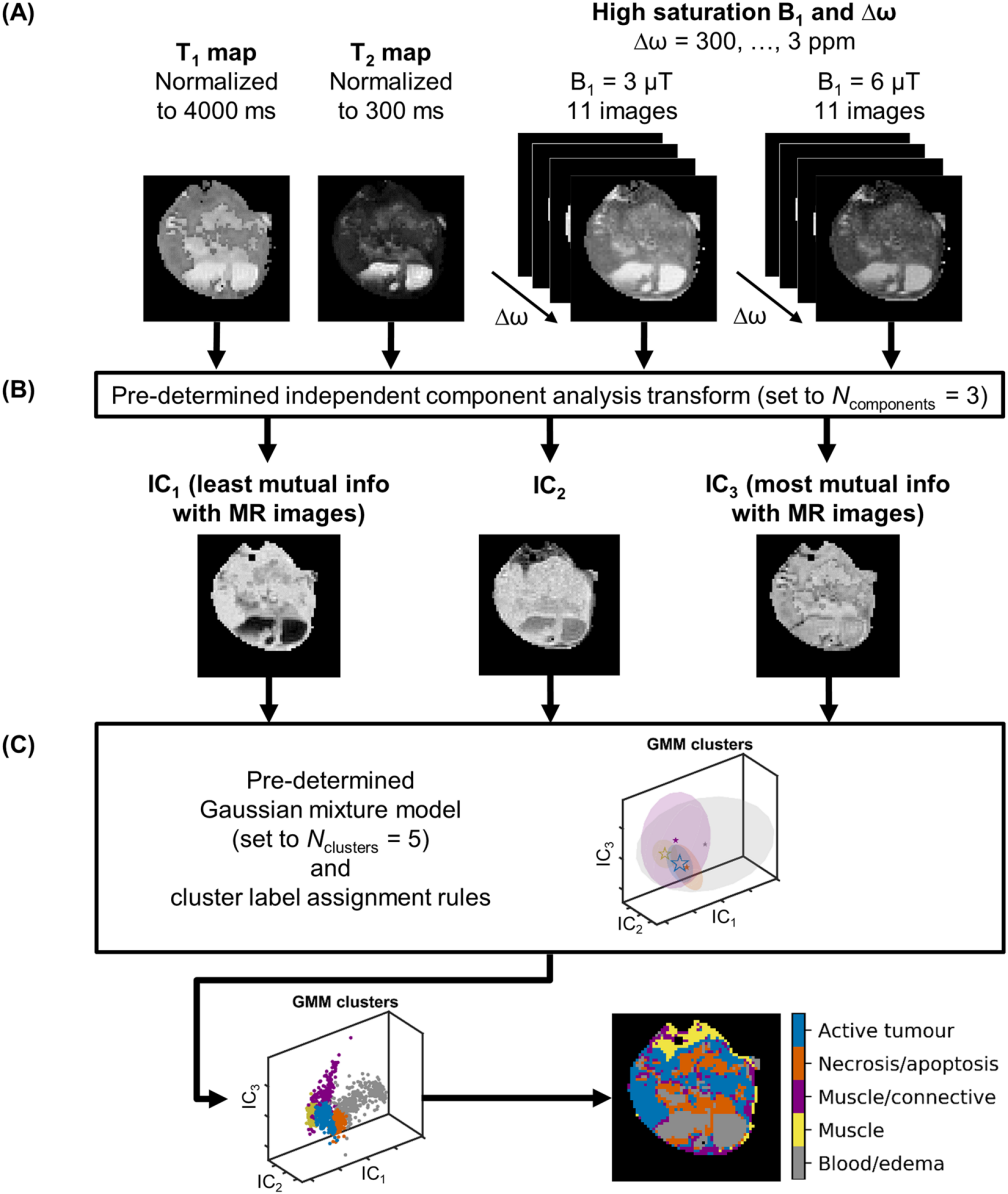
The automatic segmentation pipeline. (**A**) Normalized T_1_ and T_2_ maps and Z-spectrum images acquired with various saturation B_1_ amplitudes and at various frequency offsets, Δω (3 ppm shown). The T_1_ and T_2_ maps are normalized to values selected as being slightly higher than the highest values typically seen in tumour regions. (**B**) Non-background voxels are concatenated into an observation matrix and transformed by a trained independent component analysis transform, which is set to generate three independent component (IC) images. The ICs are sorted in order of increasing mutual information with respect to the input. (**C**) The ICs are then input to a trained Gaussian mixture model (GMM), which is set to five clusters, and the clusters are assigned labels using a pre-defined ruleset.

The segmentation pipeline (Fig. 2) consisted of running an independent component analysis^34^ on the input dataset, followed by fitting a Gaussian mixture model (GMM)^35^, assigning cluster labels based on comparison with histology, and generating a segmentation mask of the original image data. The optimal number of GMM clusters was determined using the gradient of the Bayesian information criterion^36^, calculated multiple times using unique sets of input images and spanning a range of GMM clusters (up to 10). The gradient of the BIC approached zero after five clusters and remained near zero with increasing numbers of clusters, indicating that this model can reasonably estimate five clusters (Supplementary Fig. S2).

**Figure 3:**
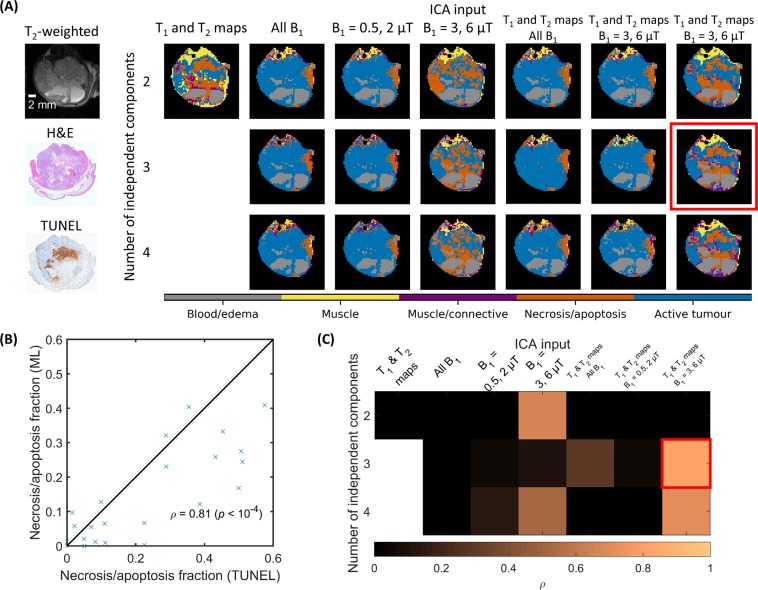
Selection of the ICA input and number of independent components. (**A**) A T_2_-weighted anatomical image and histological sections with H&E staining for general tissue discrimination and a TUNEL assay for necrosis/apoptosis are shown in the first column. Clusters calculated with various ICA inputs and numbers of independent components (ICs) are in subsequent columns. Masks were not generated with T_1_ and T_2_ maps as ICA input using three and four ICs because the number of unique image types must be equal to or greater than the number of ICs. The Gaussian mixture model was set to five clusters in all cases. The cluster label assignment is arbitrary at this stage. (**B**) Comparison of necrosis/apoptosis fractions calculated from TUNEL and machine learning (ML) using T_1_ and T_2_ maps and B_1_ = 3 and 6 µT as ICA input and three ICs (indicated by the red box in A) for all 24 mice with TUNEL assays. The line of identity and Pearson correlation coefficient ρ are also displayed. (**C**) Correlation coefficients for the ICA inputs and numbers of ICs in A. Segmentation masks from the optimized protocol with T_1_ and T_2_ maps and B_1_ = 3 and 6 µT with three ICs had the highest correlation coefficient. Only this protocol and number of ICs was considered in the remainder of this work.

Segmentation masks were generated for each imaging protocol and number of independent components (ICs) ranging from two to four. For each mask, the fraction of intratumoural voxels identified as necrosis/apoptosis was calculated, and compared with that derived from histology. The comparison of various imaging protocols and different numbers of ICs with anatomical MRI images and histological sections for the complex tumour example is shown in Fig. 3A. Spatial proportions of the histological sections did not correspond exactly to the MRI due to tissue processing and mounting. The highest Pearson correlation coefficient (ρ = 0.81, *p* < 10^−4^) was found for the imaging protocol consisting of T_1_ and T_2_ maps, and saturation transfer-weighted images with B_1_ = 3 and 6 µT and three ICs (Fig. 3B,C; see Supplementary Fig. S3 for segmented TUNEL histopathology). Therefore, only this input and number of ICs was considered in the remainder of this work and is referred to as the “optimized protocol”.

### Cluster label assignment

**Figure 4:**
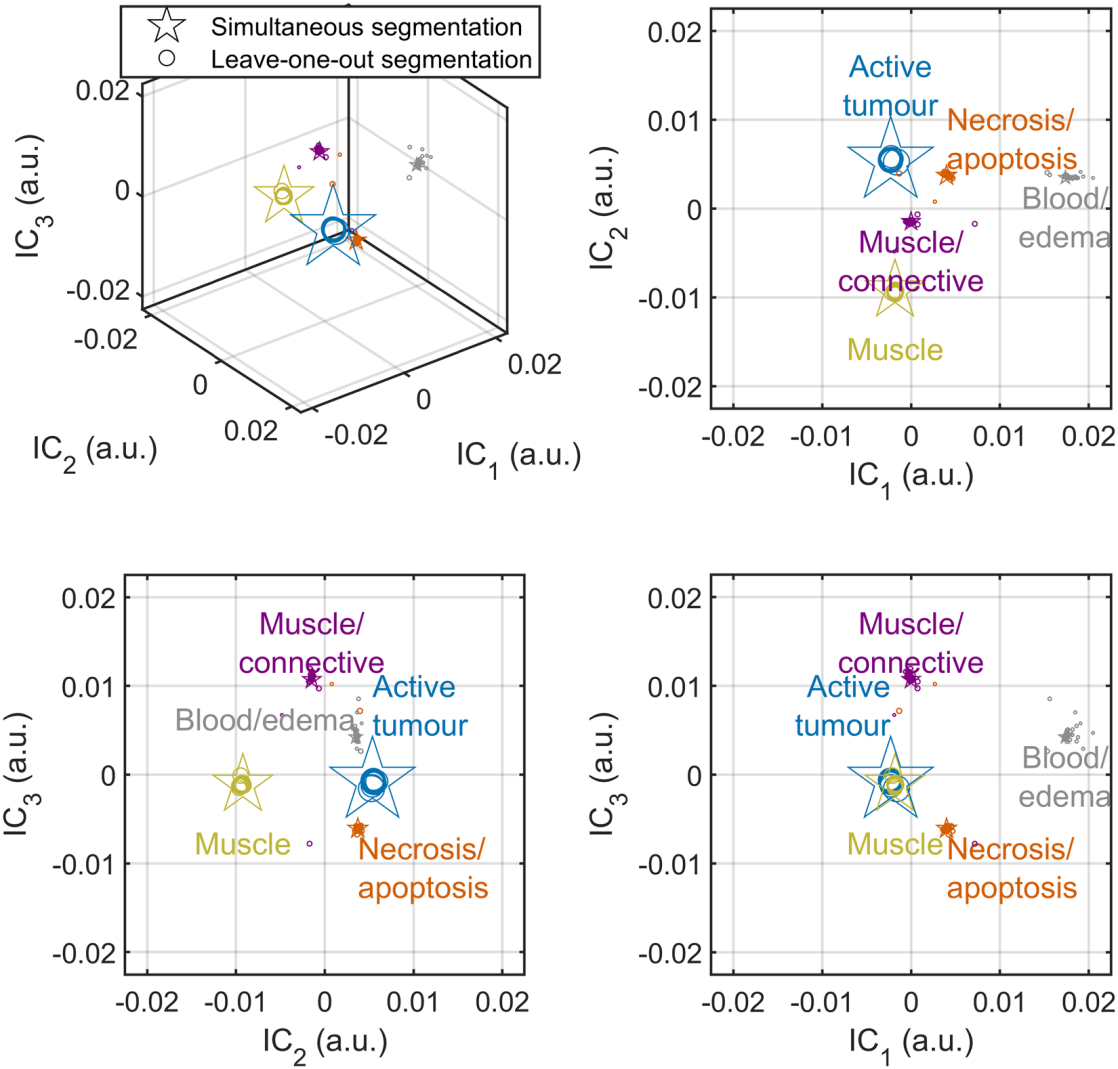
Gaussian mixture model output. Gaussian mixture model cluster means in the 3D space defined by the three independent components (ICs) when performing simultaneous segmentation on all datasets (stars; 34 mice) and leave-one-out segmentation on unique sets of 33 mice (circles) are plotted. The clusters show tight groupings, which indicate robust performance in leave-one-out cross validation. The marker size is scaled by cluster weight (circles), or 4× cluster weight (stars). For improved visibility, the variances of the Gaussians are not shown.

Figure 4 shows the labelled GMM means (stars) for the simultaneous segmentation of all 34 mice and GMM means for 34 leave-one-out segmentations (circles; 33 mice each). Of the leave-one-out segmentations, only one resulted in substantially different clusters. A graphical explanation of the label assignment algorithm can be also found in Supplementary Fig. S4.

### Robustness of segmentation pipeline

The Dice similarity coefficient between whole-dataset and leave-one-out segmentation was 98 ± 3% (mean ± SD across all mice). Leave-one-out segmentation differed greatly for one of the mice, which had a coefficient of only 84%. In this case, the necrosis/apoptosis voxels from simultaneous segmentation were erroneously added to the active tumour cluster. This is likely due to the proximity of active tumour and necrosis/apoptosis voxels in IC space, which confounded the Gaussian mixture model fitting.

**Figure 5:**
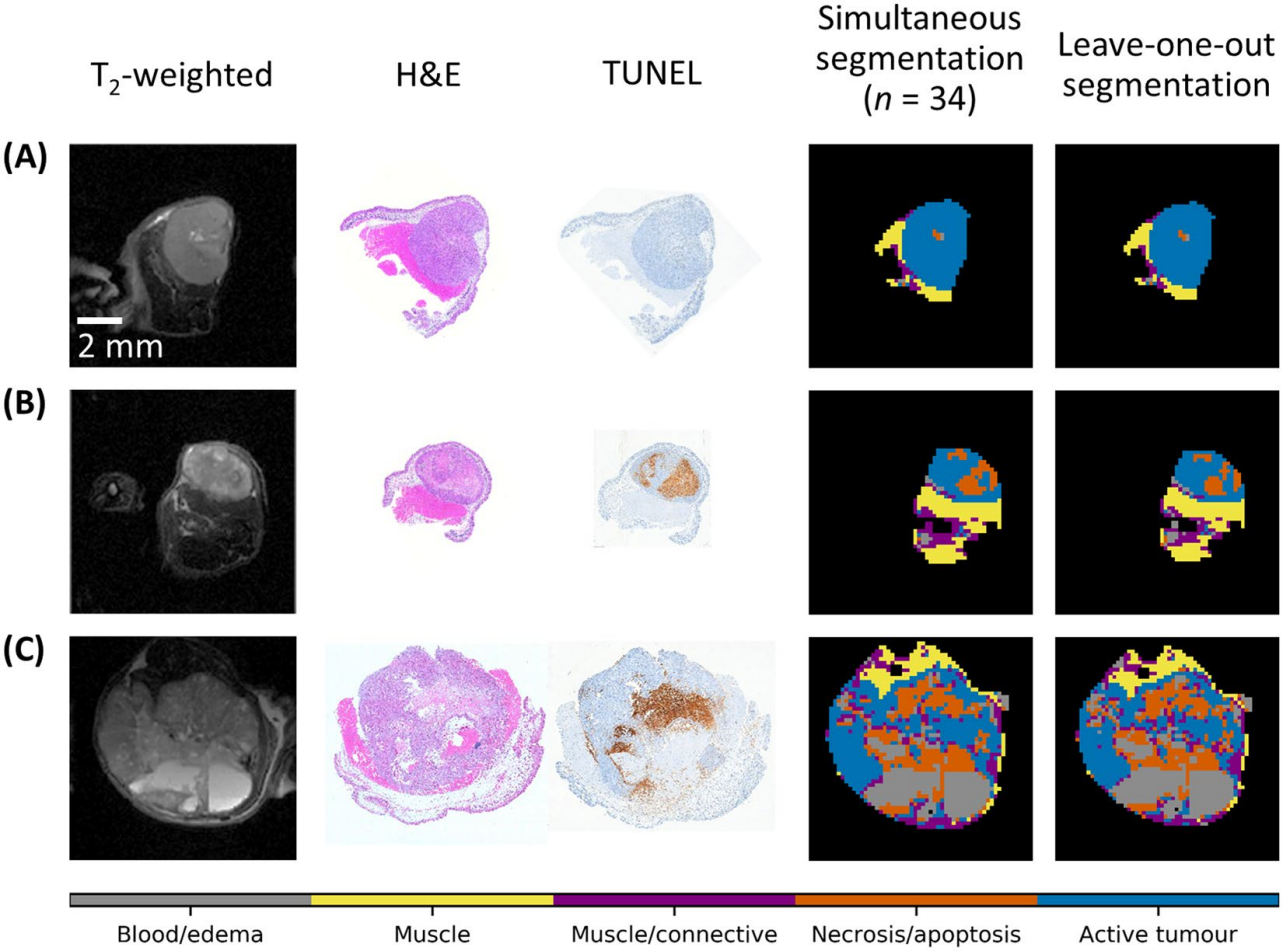
Comparison of whole-dataset and leave-one-out segmentation with anatomical images and histology for three representative cases. The tumours are (**A**) primarily active tumour; (**B**) active tumour and necrosis/apoptosis; (**C**) and active tumour, necrosis/apoptosis, and blood/edema. The leave-one-out segmentation (fifth column) was conducted using all data but the tumour shown, and the results of the segmentation were then applied to this tumour. In these cases, the morphology and extent of the brown areas indicating necrosis/apoptosis in the TUNEL sections (third column) qualitatively match with orange areas in the whole-dataset and leave-one-out segmentation masks (fourth and fifth columns, respectively). The extent of the necrosis/apoptosis in the fourth column is slightly greater than that indicated by TUNEL (third column), possibly due to the 1 mm imaging slice capturing more necrosis than the 5 µm histopathological section. A similar figure containing all the tumours can be found in Supplementary Figure S1.

Figure 5 compares segmentation performed with the whole-dataset and leave-one-out approaches along with anatomical images and histology for three representative cases. The tumours are (A) primarily active tumour; (B) active tumour and necrosis/apoptosis; (C) and active tumour, necrosis/apoptosis, and blood/edema. In the leave-one-out segmentation, the T_2_-weighted image, histology, and segmentation masks of the omitted mouse are shown. In these cases, the morphology and extent of the brown areas staining for necrosis/apoptosis in the TUNEL sections (third column) qualitatively match with the areas segmented in orange on the whole-dataset and leave-one-out segmentation masks (fourth and fifth columns, respectively). A similar figure containing all the tumours can be found in Supplementary Fig. S1; mouse #13, which had the low Dice coefficient on the leave-one-out segmentation, can be seen in that figure.

### Optimization of protocol via feature selection

The subsets of saturation transfer-weighted images and T_1_ and T_2_ maps which discriminated most accurately between tissue types are listed in Table 1. As expected, increasing the number of images in the analysis subset provides a better match to the full optimized protocol with 24 images in total, going from a Dice similarity coefficient of 93% with three images up to 98% with nine images. The positive predictive value (PPV) and negative predictive value (NPV) of the subsets for both active tumour and necrosis/apoptosis increase with the size of the image subset as expected. Of the three-image subset, which is the smallest allowed with three ICs, the PPV and NPV for active tumour and NPV for necrosis/apoptosis are at least 94%. Eight images are required to yield a PPV for necrosis/apoptosis of 90%, but this continues to rapidly improve, reaching 95% with nine images. The images common to most subsets were: the T_1_ and T_2_ map and saturation transfer-weighted images with B_1_ = 6 µT at Δω ≈ 48 ppm, which is highly sensitive to MT. Representative segmentation masks are shown in Supplementary Fig. S5. Qualitatively, masks calculated using image subsets are very similar to those using the full protocol.

**Table 1:** Assessment of image subsets via feature selection. Image subsets were selected by an exhaustive search using the Dice similarity coefficient (mean ± SD across all mice) between labels generated using the subset and the optimized protocol (i.e., T_1_ and T_2_ maps and all 22 saturation transfer-weighted images with B_1_ = 3 and 6 µT) as the metric. The positive and negative predictive value (PPV and NPV, respectively) of tumour and necrosis/apoptosis labels are also given with respect to those generated from all images from the optimized protocol.

No. of Images	T_1_	T_2_	3 µT				6 µT				Dice similarity coefficient (%)	Active tumour		Necrosis/apoptosis	
			3 ppm	5 ppm	30 ppm	48 ppm	5 ppm	8 ppm	48 ppm	75 ppm		PPV (%)	NPV (%)	PPV (%)	NPV (%)
3	X						X		X		93 ± 3	94 ± 4	96 ± 3	60 ± 30	99 ± 2
4	X						X	X	X		94 ±3	94 ± 5	97 ± 3	70 ± 30	98 ± 2
5	X		X			X		X	X		95 ± 2	97 ± 3	96 ± 3	70 ± 30	99 ± 1
6	X		X			X	X	X	X		95 ± 2	97 ± 3	97 ± 3	70 ± 30	99 ± 1
7	X	X	X			X	X	X	X		95 ± 2	95 ± 4	99 ± 1	70 ± 30	99 ± 2
8	X	X	X			X	X	X	X	X	97 ± 2	97 ± 3	99 ± 1	90 ± 10	99 ± 1
9	X	X	X	X	X	X	X	X	X		98 ± 1	98 ± 1	99 ± 1	95 ± 4	100 ± 1

### Quantitative MT model fitting

**Figure 6:**
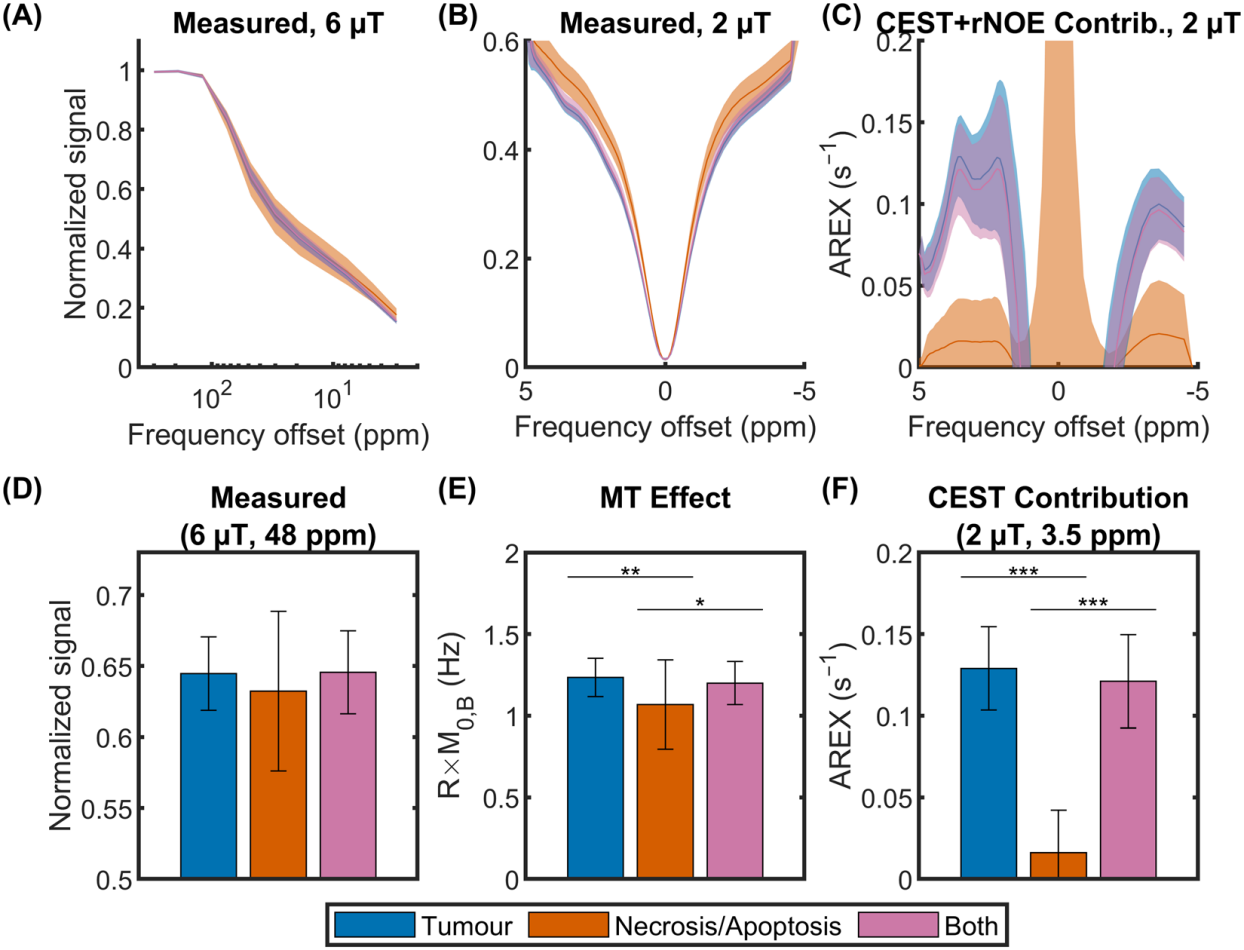
Measured saturation transfer-weighted signal and derived metrics. Measured Z-spectra and derived metrics are shown for tumour (*n* = 34), necrosis/apoptosis (*n* = 10), and combined tumour and necrosis/apoptosis (*n* = 10) regions containing at least seven voxels. Mean and standard deviation of Z-spectra with B_1_s of (**A**) 6 and (**B**) 2 µT, over all mice. (**C**) CEST and relayed-NOE contribution spectra calculated using the apparent exchange-dependent relaxation (AREX) metric, which removes the effect of T_1_. Unpaired t-test comparisons of (**D**) the MT-weighted image common to all the optimal image subsets (with B_1_ = 6 µT at 48 ppm), (**E**) MT effect, and (F) CEST contribution with B_1_ = 2 µT at the amide frequency offset (3.5 ppm). *p < 0.05. **p < 0.01. ***p < 0.001.

The observed T_1_ (*T*_1,obs_) and estimated MT model parameters for the five clusters are listed in Table 2. The product of *R* and *M*_0,B_ (fourth column), termed the MT effect, is significantly different between the active tumour and necrosis/apoptosis clusters (Fig. 6E). The blood/edema cluster (third row) has particularly large uncertainties due to its mixed composition. Representative quantitative MT model fits (used to estimate the values in Table 2) and Z-spectrum differences between clusters as a function of saturation amplitude are shown in Supplementary Fig. S6. The largest differences between interpolated MT Z-spectra of individual clusters were predicted between 37 and 62 ppm for spectra obtained at 6 µT (Supplementary Fig. S6B–G). This range corresponds well to the MT-sensitive saturation parameter set (6 µT at 48 ppm; see Table 1) that provided maximal discrimination between clusters as chosen by feature selection. Furthermore, extrapolation to higher and lower saturation powers indicates that increased B_1_ confers greater discrimination between some but not all tissues, at a frequency offset that increases with B_1_.

**Table 2:** Estimated parameters (mean ± SD) of observed T_1_ and the two-pool quantitative MT model for the five clusters. *T*_1,obs_ is the observed longitudinal relaxation time. *T*_2,A_ and *T*_2,B_ are the transverse relaxation times of the liquid and macromolecular pools, respectively. *R* is the magnetization exchange rate from the semisolid macromolecular to liquid pools. *M*_0,B_ is the macromolecular pool size relative to that of water (defined to be unity). The product of *R* and *M*_0,B_, termed MT effect, is presented because these two parameters are coupled. Blood/edema is expected to have a relatively small MT pool size^41^, which is reflected in the large uncertainties in MT effect and *T*_2,B_. All parameters were estimated for individual mice before averaging. Any given cluster per mouse was included only if it contained at least seven voxels.

Cluster	*T*_1,obs_ (ms)	*T*_2,A_ (ms)	MT Effect	*T*_2,B_ (µs)
Active tumour (*n* = 34)	2200 ± 100	53 ± 6	1.2 ± 0.1	8.2 ± 0.3
Necrosis/apoptosis (*n* = 10)	2600 ± 100	80 ± 9	1.1 ± 0.3	7.8 ± 0.2
Blood/edema (*n* = 8)	2800 ± 300	130 ± 60	0.9 ± 0.8	100 ± 100
Muscle/connective (*n* = 11)	1810 ± 80	31 ± 6	1.5 ± 0.5	7.4 ± 0.4
Muscle (*n* = 31)	1840 ± 80	27 ± 2	3.8 ± 0.5	7.2 ± 0.2

### Isolation of CEST and relayed-NOE contributions

CEST and relayed-NOE effects were calculated separately for active tumour regions, necrosis/apoptosis regions, and whole tumour (consisting of both active tumour and necrosis/apoptosis regions). The mean and standard deviation of representative high and low B_1_ Z-spectra and CEST and relayed-NOE contribution spectra over all mice for both regions plus the combined regions are shown in Fig. 6A–C. Unpaired t-test comparisons of the MT-weighted image common to all the optimal image subsets (with B_1_ = 6 µT at 48 ppm), MT effect, and CEST contribution at the amide frequency offset (3.5 ppm) with B_1_ = 2 µT for the same clusters are shown in Fig. 6D–F. There are significant differences are between the tumour and necrosis/apoptosis clusters. The values for combined tumour and necrosis/apoptosis clusters lie between those of the tumour and necrosis/apoptosis clusters as expected. This supports the requirement for separate tumour and necrosis/apoptosis clusters when analysing quantitative data.

## Discussion

In this study, an automatic framework was developed for segmenting intratumoural regions using T_1_ and T_2_ maps and saturation transfer-weighted images. The segmentation pipeline consisted of a three-component ICA transform and a five-cluster GMM which took less than one second to calculate. The optimal imaging protocol was determined to be the set of T_1_ and T_2_ maps and saturation transfer weighted images with B_1_ = 3 and 6 µT. Using histology, the five clusters were identified as corresponding to three intratumoural regions (active tumour, necrosis/apoptosis, and blood/edema) and two extratumoural (muscle and a mix of muscle and connective tissue). This automated segmentation qualitatively matched anatomical and histological images. Although there are no other studies using saturation transfer weighted MRI to automatically segment intratumoural regions to our knowledge, we discuss several relevant studies below. This will be followed by discussion of possible improvements to the pipeline. Finally, the novel use in this work of the apparent exchange-dependent relaxation (AREX) metric with an extrapolated MT reference (EMR) to isolate an aggregate CEST and rNOE contribution spectrum instead of the conventional multi-Lorentzian reference to yield individual contributions for each pool will be discussed.

Henning *et al*.^18^ identified two regions of viable tumour (normoxic and hypoxic), two of non-viable tumour in xenografts (*n* = 13) and a background region using apparent diffusion coefficient (ADC), T_2_, and proton density maps input into a k-means clustering algorithm. A significant Pearson correlation coefficient between k-means and histologically derived tumour volumes of 0.94 was found.

Jardim-Perassi *et al*.^14^ also identified the same four regions in xenografts using T_2,_ T_2_*, and ADC maps and three dynamic contrast enhanced (DCE) model parameter maps input into a GMM. Histological sections were cut while each excised tumour was placed in a tumour-specific 3D printed sectioning template, which improved co-registration between MRI and histology. The quality of fit to the histologic slices was quantified with the Jaccard index which was 82 ± 4% (*n* = 16). Chang *et al*.^20^ identified two regions of active tumour (well-perfused and hypoxic) and one of necrotic using only DCE scans of xenografts (*n* = 1 prostate and *n* = 2 brain tumours). The clustering was performed based on the area under the contrast agent wash-in and wash-out curve. They showed a prostate xenograft histological section, which had some overlap with the segmentation mask, but also notable differences. However, these were not quantified. These two protocols required the injection of a gadolinium-based contrast agent to generate DCE images, which is contraindicated in renally impaired patients^31^ and the background appeared to be removed manually.

Katiyar *et al*.^17^ identified three regions: viable, necrotic, and peri-necrotic using T_2_-weighted images, ADC maps, and pre- and post-contrast T_2_ and T_2_* maps of xenografts (*n* = 6) input into several clustering methods. The method that performed the best was spatially regularized spectral clustering, which yielded Pearson correlation coefficients of 0.98, 0.92, and 0.82 for the three regions, respectively. The contrast agent was an injected superparamagnetic iron oxide nanoparticle approved to treat iron deficiency anaemia. However, it is unsuitable for frequent use because alteration of MRI imaging studies may persist for up to three months^37^. This group also published a method^15^ to identify necrotic and viable voxels using an ADC map and ^18^F-FDG positron emission tomography image in xenografts (*n* = 4) input into a 2D GMM. Pearson correlation coefficients of 0.87 and 0.88, respectively, were found between histology and clustering. In this case, the use of ionizing radiation is undesirable. The background also appeared to be removed manually.

Alignment of histopathology with MRI and segmentation masks was a significant challenge and is an area of active research. Determination of complex tumour histopathology requires interpretation by a clinical radiologist and/or pathologist, which can produce variant annotation between observers^38^. There is disproportionate image resolution between a large MRI voxel, capturing greater tissue heterogeneity, versus the cellular composition represented by histology^39^. Furthermore, contrast achieved in MRI may not easily align with variations of chromogen staining in tissue. Shape and orientation of the tissue depends on positioning of the body part in MRI. Similarly, excision of the tumour, tissue fixation causing dehydration, and a shift in the slicing plane through the tissue, add to the complexity aligning size and shape^39^. The mismatched uniformity between the two techniques, along with subjective interpretation of images, facilitates error in anatomical and pathological measurements.

The choice of ICA over principal component analysis (PCA)^40^, another commonly used dimensionality-reduction technique, is logically based on ICA’s assumption of independent sources. PCA, on the other hand, tries to find components that explain maximum variance drawn from across all clusters. Segmentation of this dataset using PCA resulted in more overlap between clusters, although the segmentation masks generated were largely similar (data not shown).

For the necrosis/apoptosis cluster, the positive predictive value (PPV) of segmentation masks generated from the three- to seven-image subsets was below 90%. It may be possible to increase this by choosing another metric for feature selection. The metric used in this work was the Dice similarity coefficient between image-subset and whole-dataset segmentation. However, a metric which explicitly includes for the PPV of necrosis/apoptosis cluster may increase this PPV for smaller image subsets.

The large degree of uncertainty in the estimated parameters of the blood/edema cluster are likely due to different sources. For the observed T_1_ (*T*_1,obs_) and T_2_ of the free water pool (*T*_2,A_), the source is probably both mixed composition and the relatively small number of samples. As an example, the regions with the two highest coefficients of variation (SD/mean) of *T*_1,obs_ are muscle/connective tissue and blood/edema (19% and 15%, respectively) are the smallest regions, representing 7% and 2% of all voxels, respectively. The uncertainty in the MT effect (*R* × *M*_0,B_) and T_2_ of the MT pool (*T*_2,B_) is probably due the fact that blood/edema is expected to have little MT effect^41^.

There is further evidence that the blood/edema cluster is of mixed composition and could be sub-clustered. A scatter plot of the blood/edema voxels in the complex tumour in Fig. 2 as a function of their observed T_1_ and T_2_ shows the presence of a possible second cluster (Supplementary Fig. S7). The larger cluster, probably edema, is between the literature values of blood, cerebrospinal fluid, and synovial fluid. The second cluster contains far fewer voxels scattered around the observed T_1_ and T_2_ of blood. However, there was an insufficient number of these voxels in our dataset to train the model to detect a sixth cluster.

Although feature selection and quantitative MT modelling indicated that 48 ppm was the frequency offset giving the largest contrast differences between clusters, the inclusion of images at 5–8 ppm by feature selection could be due to sensitivity to CEST that these offsets provide, since necrotic and apoptotic cells are expected to have decreased metabolism to which CEST is sensitive. Overall, however, MT contrast appears to better inform the segmentation algorithm presented than does CEST contrast. Note that MT modelling is not necessary for the segmentation algorithm presented in this work.

To evaluate the CEST contribution, we used the EMR^42^ technique, but added, for the first time to our knowledge, the AREX metric^43,44^ in order to remove the effects of T_1_. The original multi-pool AREX method requires fitting a summed-Lorentzian model^45^ to one low B_1_ Z-spectrum. Then, one Lorentzian spectral contribution for each pool is extracted using the sum of the other modelled pools’ signals as a baseline^46^ (called the reference Z-spectrum *Z*_ref_) and an observed T_1_ map. The adapted method, used here, extracts only one spectrum, with the contributions of all CEST and rNOE pools in aggregate, using the EMR Z-spectrum as *Z*_ref_. This is potentially faster because the number of measurements required to generate an EMR *Z*_ref_ is much less than a low B_1_ *Z*_ref_ (22 offsets at high B_1_ and a T_1_ map vs 66 offsets, respectively, in this work). Note that, using the EMR as the reference spectrum, measurements at low B_1_ only need to be made at the offset(s) where one wishes to assess the CEST or rNOE contribution. The aggregate CEST and rNOE contribution spectrum contains contributions from multiple chemical groups at each offset, but requires less data acquisition and, unlike a summed-Lorentzian model, doesn’t assume a fixed number of pools.

Future work will incorporate multi-slice imaging enabling through-plane registration, adding an intermediate *ex vivo* MRI scan, creating a tissue sectioning template, and developing 3D imaging techniques for histopathology such as whole tumour slice reconstruction^14,38,39^. Together these will provide quantification of whole tissue and sub-regional detail for improved alignment and translation between MRI and histopathology. Another possible modification to the segmentation pipeline would be to introduce an individual weighting for each IC before input to the GMM. This is left for future work because, when a grid search of the correlation of necrosis fraction from machine learning and histology as a function of IC weight (up to 9, 16, and 16 for IC_1_, IC_2_, and IC_3_, respectively), no trend was apparent. Although there was a local maximum in correlation (ρ = 0.90 with an IC_1_:IC_2_:IC_3_ weighting of 1:3:2 compared to 0.81 with a weighting of 1:1:1 as shown in Fig. 3B), it was decided not to weight the ICs in this work because there was no logical rationale to do so.

## Methods

### Animal model

Approximately 3 × 10^6^ DU145 human prostate adenocarcinoma (ATCC, Manassas, VA) cells mixed in a 1:1 ratio by volume with growth factor reduced Matrigel matrix (BD Canada, Mississauga, ON) were injected in the right hind limbs of 34 female athymic nude mice (Charles River Canada, Saint-Constant, QC) and allowed to grow into tumours for at least 34 days post-injection. Tumours were measured using callipers every one to four days and their volume was calculated using the formula volume = length × width^2^/2. All experimental procedures were approved by the Animal Care Committee of the Sunnybrook Research Institute, which adheres to the Policies and Guidelines of the Canadian Council on Animal Care and meets all the requirements of the Animals for Research Act of Ontario and the Health of Animals Act of Canada.

### Magnetic resonance imaging

Tumours were scanned at 7T (BioSpec 70/30 USR with BGA-12SHP gradients running ParaVision 6.0.1, Bruker BioSpin, Billerica, MA) using an 86 mm inner diameter volume coil for transmit and a 20 mm diameter loop surface coil for receive. A fifteen-slice 2D axial T_2_-weighted rapid acquisition with refocused echoes^47^ (RARE; TR  =  2500 ms; TE_eff_  =  55 ms; FOV  =  20 mm × 20 mm; slice thickness = 0.5 mm; matrix = 128 × 128; RARE factor = 12; bandwidth = 33 kHz; averages = 4; 6 min, 40 s) was used for prescribing the slice of interest, chosen to be at the thickest point of the tumour. B_0_-map-based shimming (MapShim) of second order gradients was performed on an ellipsoidal volume enclosing the tumour in the slice of interest. Flip angle scale factor maps^48^ were calculated for the first four mice using a series of 3D high flip angle fast low angle shot (FLASH)^49^ scans and the T_1_ map for the slice of interest and the flip angle in the tumour region of interest was found to be within 6% of nominal (Supplementary Fig. S7 in our previous work^23^). Thus, B_1_ correction was deemed unnecessary going forward.

Saturation transfer-weighted images were acquired using a 490 ms block RF saturation pulse per k-space line and single-slice FLASH acquisition (TR = 500 ms; TE   =  3 ms; flip angle = 30°; FOV = 20 mm × 20 mm; slice thickness = 1 mm; matrix = 64 × 64; bandwidth = 50 kHz; and 1 dummy scan) as in our previous work^45^. The cumulative saturation time when acquiring the centre of k-space was approximately 16 s. Five datasets were acquired: two Z-spectra sensitive to the direct water saturation effect (DE), CEST, and MT with B_1_ = 0.5 and 2 µT at 66 frequency offsets Δω = (ω − ω_0_)/ω_0_ × 10^6^ (where ω is the saturation frequency and ω_0_, the water resonance frequency) between ±5 ppm; two Z-spectra mainly sensitive to DE and MT with B_1_ = 3 and 6 µT at 11 logarithmically spaced Δω between 300 and 3 ppm; and one WASSR Z-spectrum^50^ sensitive only to DE with B_1_ = 0.1 µT at 21 Δω between ±0.5 ppm.

To allow for correction of system instability in post-processing, reference scans at Δω = 667 ppm were acquired before and after and also interleaved between every five Z-spectrum measurements^23,45^. The scan time for the Z-spectra including reference scans with B_1_ = 0.5 and 2 µT was 44 min/spectrum; 3 and 6 µT, 8.5 min/spectrum; and 0.1 µT, 15 min. To evaluate longitudinal relaxation time T_1_, five inversion recovery RARE scans (TR = 10,000 ms; TE_eff_ =  10 ms; TI = 30, 110, 390, 1400, 5000 ms; same FOV, slice thickness, and matrix as FLASH; RARE factor = 4; bandwidth = 77 kHz; 2 min each) were also acquired for a T_1_ map^51^. The total acquisition time including scout and shimming was 2.5 h per animal.

### Histopathology

Tumours were excised for histopathological assessment immediately after scanning. Each tumour was isolated and marked with a suture on the proximal margin for subsequent alignment with MRI, formalin fixed for 24 to 48 h, and then stored in 70% ethanol until submitted for further processing. Tumours were trimmed for sectioning in the region that corresponded as closely as possible to the MRI slice. Tissues were paraffin embedded, sectioned at 10 µm, and mounted on slides. Two types of histological section were prepared: H&E staining for structural detail and a TUNEL assay using 3,3’-diaminobenzidine (DAB) chromogen and haematoxylin counter staining for necrosis/apoptosis. The tissue section that best correlated with the MRI slice was imaged using an Axio Imager 2 (version M2, Carl Zeiss Canada Ltd., Toronto, ON) microscope with the Stereo Investigator (MBF Bioscience, Williston, VT) stereology system.

### MRI data pre-processing

For each animal, images were registered using the *imregister* function in MATLAB, which was set to rigid body transform, with the first Z-spectrum reference image acquired with B_1_ = 0.5 µT as the registration reference image. In order to avoid misregistration of low SNR images acquired with saturation near the water resonance, Z-spectrum images with less than 50% of the mean signal of the reference scan were registered using the transformation matrix of the last image with sufficient SNR, typically an interleaved reference scan. Baseline drift correction of all Z-spectrum scans consisted of fitting a straight line to the interleaved reference scans. This was followed by spectrum-wise B_0_ correction of the WASSR and Z-spectrum images with low B_1_ (0.5 and 2 µT). The correction consisted of fitting one Lorentzian (corresponding to the DE contribution) to the WASSR Z-spectrum at frequency offsets between ±0.5 ppm and a sum of two Lorentzians (corresponding to the DE and MT contributions) to the low B_1_ Z-spectra. The spectra were re-centred to the peak position of the DE Lorentzian and linearly interpolated to the frequency offsets measured originally. High B_1_ images (MT sensitive) were acquired with logarithmically spaced offsets ranging from 3 to 300 ppm. Thus, B_0_ correction was not required for these spectra.

A T_1_ map was calculated from the inversion recovery scans by fitting to the inversion recovery RARE signal equation^51^. Then, a T_2_ map was calculated from the T_1_ map and WASSR Z-spectrum using the steady-state direct water saturation signal intensity (as in previous work^23^):

1 $$S(\Delta \omega )={S}_{0}\frac{{R}_{1}[{R}_{2}^{2}+{\{\Delta \omega \}}^{2}]}{{R}_{1}[{R}_{2}^{2}+{\{\Delta \omega \}}^{2}]+{\omega }_{1}^{2}{R}_{2}},$$

where R_1_ = 1/T_1_, R_2_ = 1/T_2_, and ω_1_ = γB_1_. T_1_ and T_2_ values were normalized by 4000 and 300 ms, respectively, which were values selected as being slightly higher than the highest values typically seen in tumour regions to match the range of the saturation transfer images prior to segmentation.

The pre-processing above was performed in MATLAB (Release 2019a, The MathWorks, Natick, MA). Subsequent processing was performed in Python (version 3.7) with the SciPy (version 1.4.1) scientific computing, OpenCV (version 3.4.1) computer vision, and scikit-learn (version 0.22.1) machine learning libraries.

Image erosion was used to remove edge voxels, which can be contaminated by partial volume effects. Erosion was performed for all masked images using the *binary_erosion* function in SciPy using a rank 2 structuring element where all elements are neighbours.

### Optimization of segmentation pipeline

In order to determine the optimal imaging protocol to accurately segment the tumours, seven different protocols were compared: (1) T_1_ and T_2_ maps only, (2) all Z-spectrum images, (3) Z-spectrum images acquired at low B_1_ (0.5 and 2 µT), (4) Z-spectrum images acquired at high B_1_ (3 and 6 µT), (5) T_1_ and T_2_ maps and Z-spectrum images acquired at high B_1_ (3 and 6 µT), (6) T_1_ and T_2_ maps and Z-spectrum images acquired at low B_1_ (0.5 and 2 µT), and (7) T_1_ and T_2_ maps and all Z-spectrum images. The segmentation pipeline is shown as a flowchart in Fig. 2 and was applied to all seven protocols.

For each of the seven protocols, the images were used to generate an observation matrix. The number of rows *m* is the total number of voxels across all animals and given by:

2 $$m=\mathop{\sum }\limits_{i=1}^{p}{q}_{i},$$

where *p* is the number of mice and *q*_*i*_ the number of voxels in mouse *i*. The number of columns *n* is the number of contrast types (i.e., T_1_ map, T_2_ map, and saturation transfer-weighted images) and varied with protocol.

For each observation matrix, an ICA was performed using the FastICA algorithm^34^. ICA is a linear transformation from the original feature space to a new one such that the new features are mutually independent (Fig. 2B). Transformation into two to four independent components (ICs) was investigated. One of the ambiguities with ICA is the order of the ICs. In this study, the ICs of each dataset were sorted in order of increasing mutual information between each component and the average of all protocol images, calculated using the *normalized_mutal_info_score* function in scikit-learn normalized to the arithmetic mean of the ICs and average images, and labelled IC_1_, IC_2_, and so forth.

A GMM^35^ is a probabilistic model that identifies clusters with Gaussian distributions within the multi-dimensional IC space, where the number of dimensions corresponds to the number of ICs (*N*_ICs_). Figure 2C shows clusters in 3D IC space. Each cluster had a weighting, mean (in *N*_ICs_ dimensions), and full covariance matrix (i.e., each Gaussian may adopt any position and shape). To determine the optimal number of clusters, a GMM was fitted using one to ten clusters. The optimal number of clusters for each protocol was computed using the gradient of the Bayesian information criterion (BIC)^36^ as the metric. A lower BIC indicates a greater goodness of fit, but it decreases more slowly when more than the optimal number of clusters is used. However, the point at which this happens is difficult to discern. Instead, the gradient of the BIC with respect to the number of clusters was calculated to give a function that sharply approaches zero until the optimal number of clusters and remains relatively constant afterwards. This point is straightforward to determine visually. Once the optimal number of clusters was determined, the GMM was re-fitted three times with two, three, and four ICs.

The optimal imaging protocol was selected by quantitatively comparing the necrosis/apoptosis fraction, which was defined as the number of necrosis/apoptosis voxels normalized by the sum of the number of tumour and necrosis/apoptosis voxels, calculated from the proposed pipeline (where regions were manually assigned) and from histopathology. The latter was based on TUNEL histopathology images for all 24 mice with TUNEL assays. First, the muscle and skin voxels were manually cropped out of the TUNEL images with the aid of the H&E sections and then the blue channel of the cropped RGB image was thresholded. Voxels with blue channel values below 0.78 were classified as tumour and above or equal to 0.78 as necrosis/apoptosis. The segmented TUNEL images are shown in Supplementary Fig. S3. The comparison was performed using the Pearson correlation coefficient.

### Cluster label assignment

Following the optimization of the imaging protocol, histopathology was used to inform the following label assignment algorithm: 1) the cluster with the largest absolute value of the GMM mean of IC_1_ was labelled blood/edema; 2) each dataset was reflected about the IC_1_ = 0, IC_2_ = 0, …, ${{\rm{I}}{\rm{C}}}_{{N}_{{\rm{I}}{\rm{C}}{\rm{s}}}}$ = 0 planes, as required, such that the blood/edema cluster was in the first quadrant (if ICA space is 2D), octant (if ICA space is 3D), and so forth, since ICA does not identify the sign of the source signals; 3) of the remaining clusters, the one with the smallest (i.e., most negative) GMM mean of IC_2_ was labelled muscle; the second smallest, muscle/connective; the second largest, necrosis/apoptosis; and the largest, active tumour. Supplementary Fig. S4 shows this graphically.

### Robustness of segmentation

The robustness of the segmentation pipeline was tested using leave-one-out cross-validation, where the model was trained using 33 of the 34 datasets and the trained model was applied to the final (testing) dataset. Training consisted of the generation of the ICA basis set, determining the order of the ICs, calculation of GMM clusters, and assigning labels to the clusters. The segmentation of the testing dataset, which was not part of the training dataset, was then quantitatively compared with the original segmentation using the Dice similarity coefficient.

### Optimization of protocol via feature selection

In order to reduce the number of images required for segmentation, an exhaustive search of the combinations of two to nine different images was performed and the clusters were compared with those generated using the full optimal protocol. The metric used was the Dice similarity coefficient between the voxel labels generated from the original and reduced datasets. When the optimal subsets were found, the positive and negative predictive values (PPV and NPV, respectively) of each subset for tumour and necrosis/apoptosis voxels was calculated relative to the clusters generated using the full protocol. The difference between the measured signal at the MT-weighted image common to all the optimal image subsets between tumour, necrosis/apoptosis, and combined tumour and necrosis/apoptosis clusters over all mice were compared using unpaired t-tests.

### Quantitative MT model fitting

Quantitative MT model fitting was performed in order to test the results of the feature selection, as well as to determine whether a different selection of B_1_ amplitudes or frequency offsets would improve the contrast between clusters. T_1_ maps and Z-spectra with B_1_ = 0.1, 3, and 6 µT were fitted to a two-pool MT model^52^ using a super-Lorentzian lineshape for the semisolid macromolecular pool for the muscle, muscle/connective, necrosis/apoptosis, and active tumour voxels and a Lorentzian one for the blood/edema voxels^53^. All parameters were fitted for individual mice, provided there were at least seven voxels in a given cluster (which was the size of the smallest active tumour cluster), and then averaged together. This model was used to extrapolate Z-spectra over a range of B_1_ values. The difference in extrapolated Z-spectra between each possible pair of tissue types was calculated as a measure of inter-cluster contrast. The difference between the MT effect (defined as the exchange rate *R* times the MT pool size *M*_0,B_) between tumour, necrosis/apoptosis, and combined tumour and necrosis/apoptosis clusters over all mice were compared using unpaired t-tests.

### Isolation of CEST and relayed-NOE contributions

The extrapolated semi-solid magnetization transfer reference (EMR)^42^ was calculated using the MT model parameters, which represents the MT effect. The apparent exchange-dependent relaxation (AREX) metric^43,44^ to remove T_1_ effects for CEST and rNOE contributions from each tumour and necrosis/apoptosis region was calculated as follows:

3 $${{\rm{MTR}}}_{{\rm{AREX}}}=\frac{1}{{Z}_{{\rm{lab}}}}-\frac{1}{{Z}_{{\rm{EMR}}}}$$

4 $${\rm{AREX}}=\frac{{{\rm{MTR}}}_{{\rm{AREX}}}}{{T}_{1,{\rm{obs}}}},$$

where the measured Z-spectrum (B_1_s of 0.5 and 2 µT were each used) is denoted *Z*_lab_, the extrapolated MT reference is *Z*_EMR_, and *T*_1,obs_ is the measured T_1_. The difference between the mean CEST-only contribution at 3.5 ppm between tumour, necrosis/apoptosis, and combined tumour and necrosis/apoptosis clusters over all mice were compared using unpaired t-tests.

## Supplementary information

Supplementary Information.

## Data Availability

The data that support the findings of this study are available from the corresponding author upon reasonable request.
