# Supplementary material for: An Automated Segmentation Pipeline for Intratumoural Regions in Animal Xenografts Using Machine Learning and Saturation Transfer MRI
Source: Sci Rep. 2020 May 15;10:8063. doi: 10.1038/s41598-020-64912-6 (PMC7228927; doi:10.1038/s41598-020-64912-6)
Supplement: Supplementary file 1 — Supplementary Information. [file 41598_2020_64912_MOESM1_ESM.pdf]

Supplementary Information for

An Automated Segmentation Pipeline for Intratumoural Regions in Animal Xenografts Using Machine Learning and Saturation Transfer MRI

Wilfred W. Lam, Wendy Oakden, Elham Karami, Margaret M. Koletar, Leedan Murray, Stanley K. Liu, Ali Sadeghi-Naini, Greg J. Stanisz

Note: Figure S1 spans several pages and starts below.

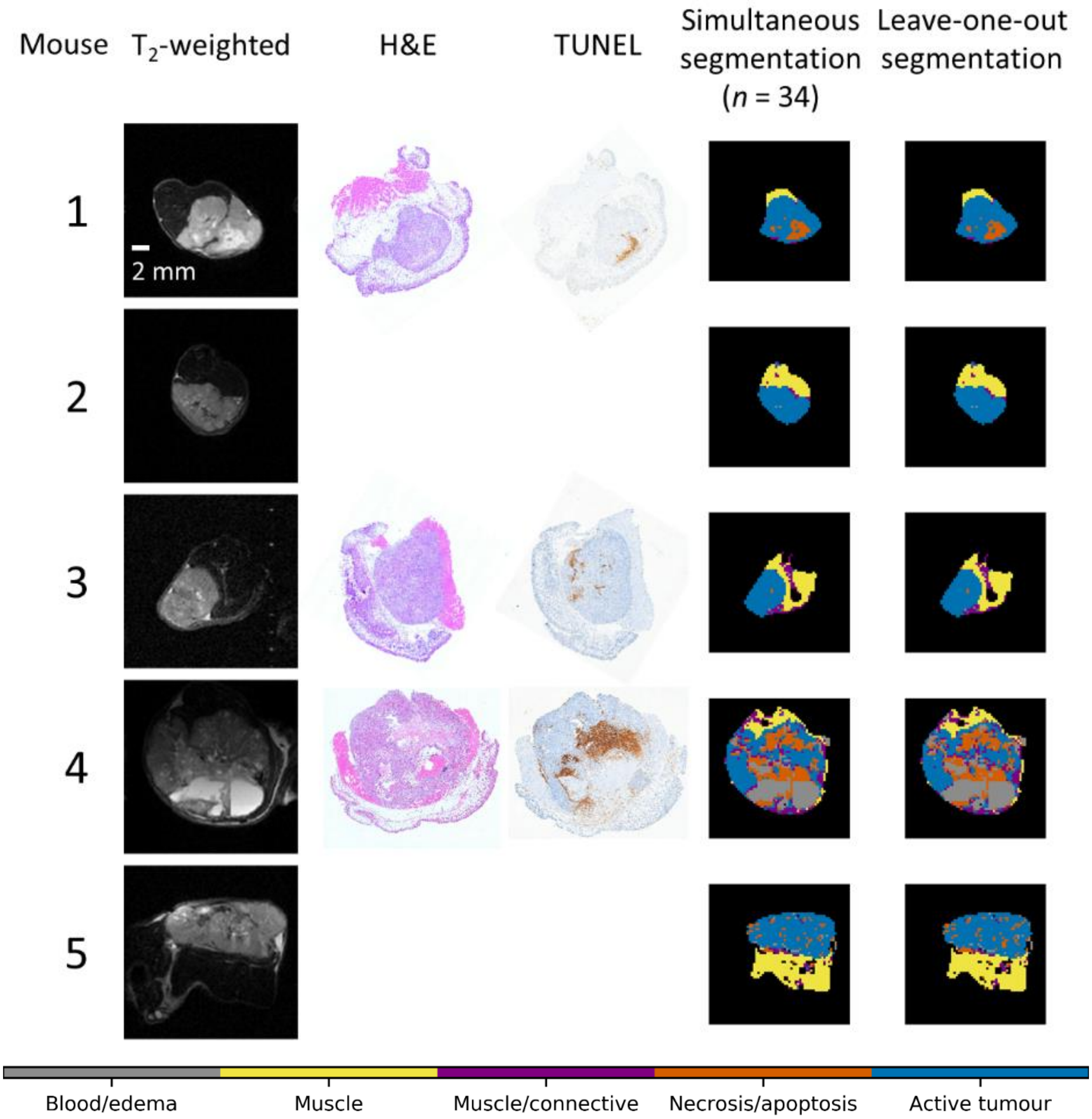

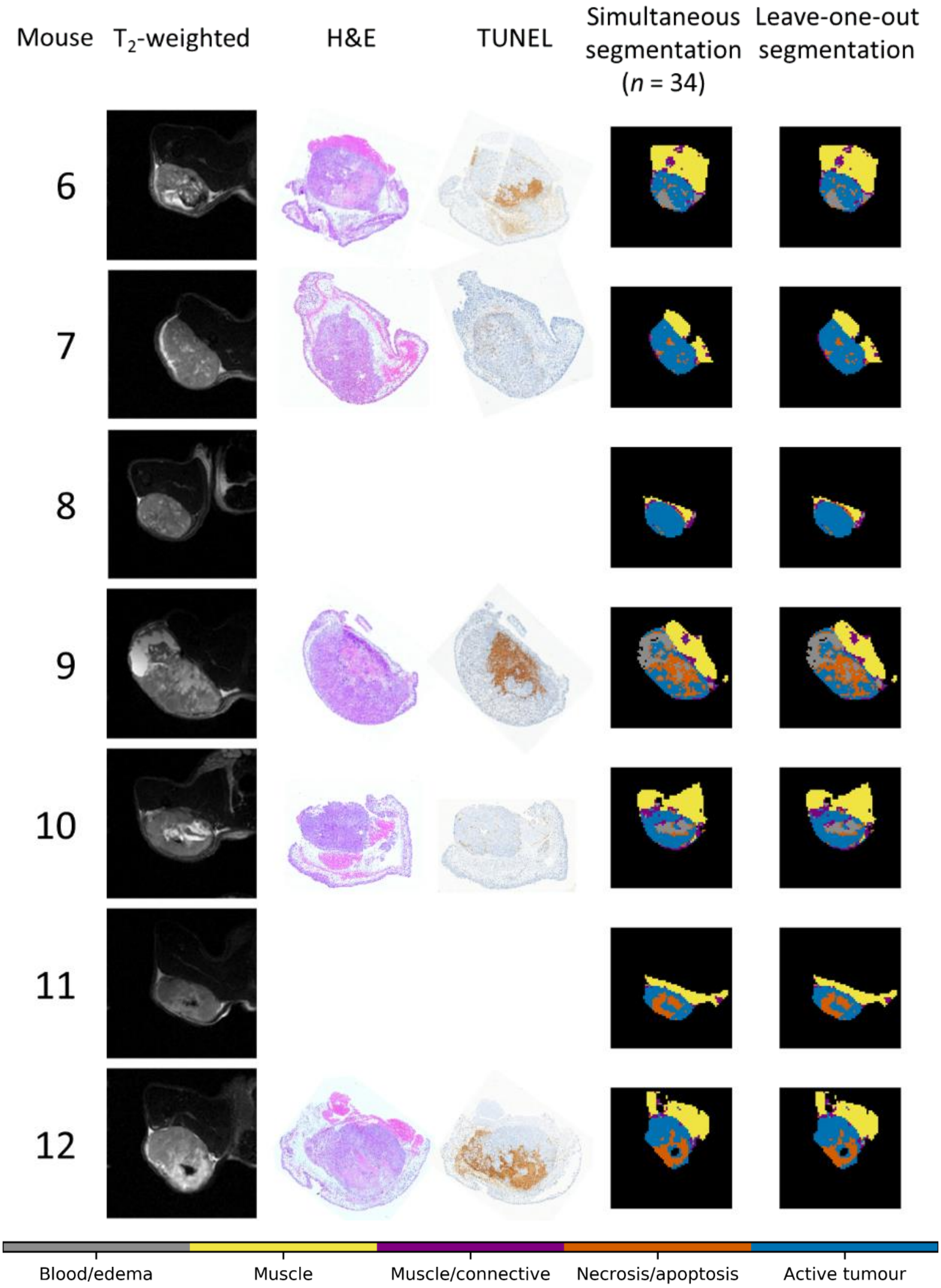

Mouse
T2-weighted
H&E
TUNEL
Simultaneous segmentation (n = 34)
Leave-one-out segmentation
6
7
8
9
10
11
12
Blood/edema
Muscle
Muscle/connective
Necrosis/apoptosis
Active tumour

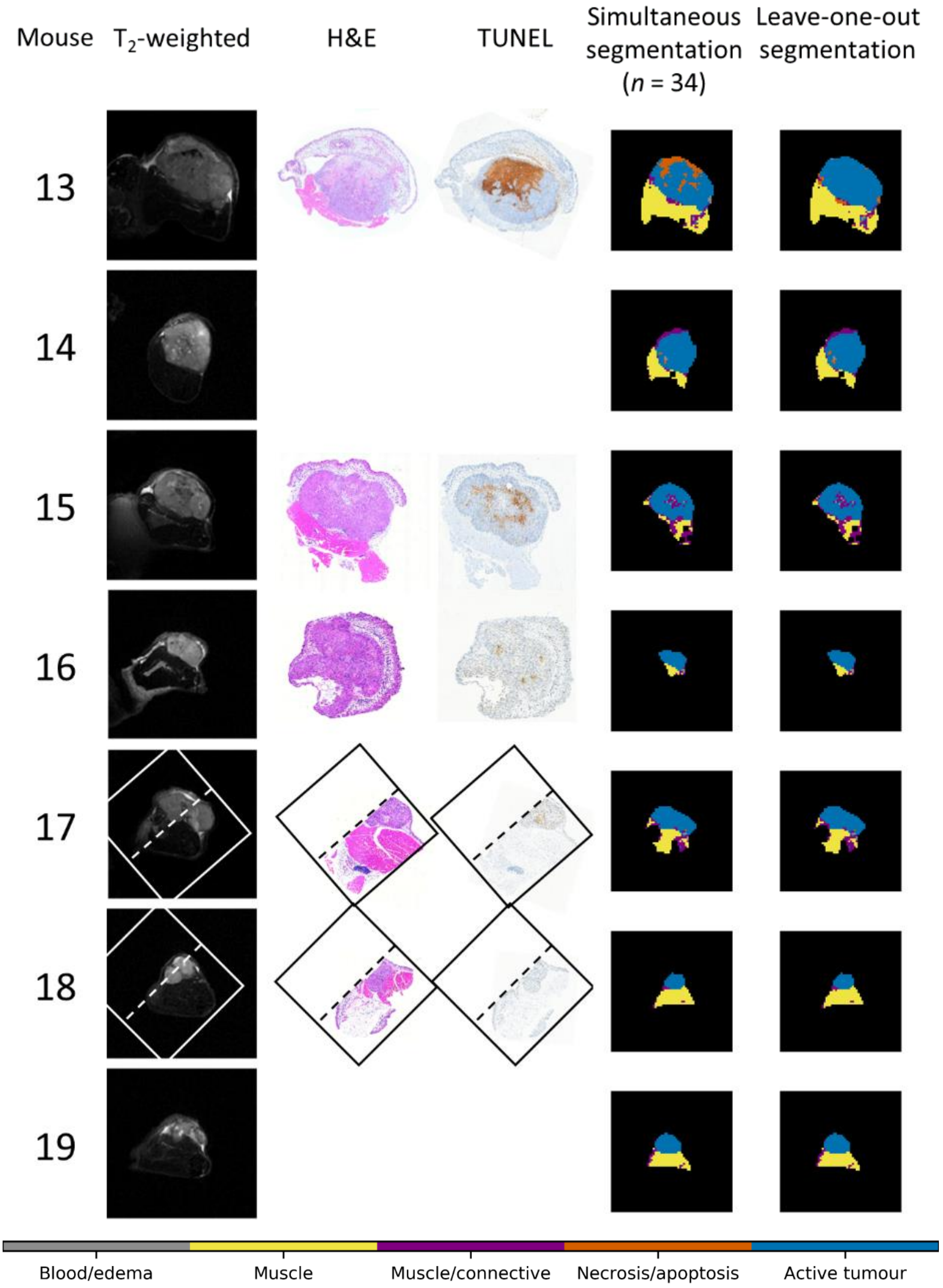

Mouse
T2-weighted
H&E
TUNEL
Simultaneous segmentation (n = 34)
Leave-one-out segmentation
13
14
15
16
17
18
19
Blood/edema
Muscle
Muscle/connective
Necrosis/apoptosis
Active tumour

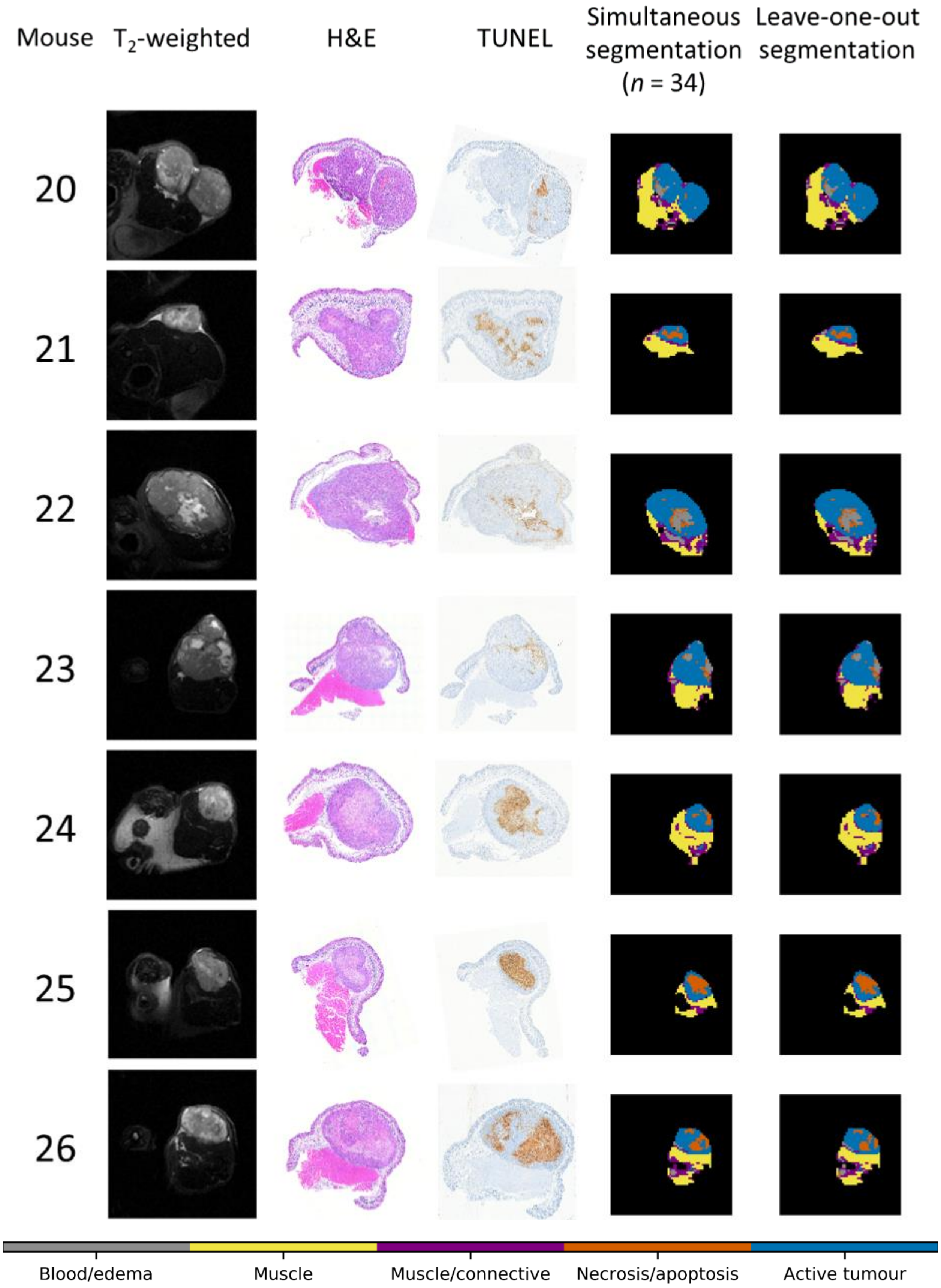

Mouse
T_2-weighted
H&E
TUNEL
Simultaneous segmentation (n = 34)
Leave-one-out segmentation
20
21
22
23
24
25
26
Blood/edema
Muscle
Muscle/connective
Necrosis/apoptosis
Active tumour

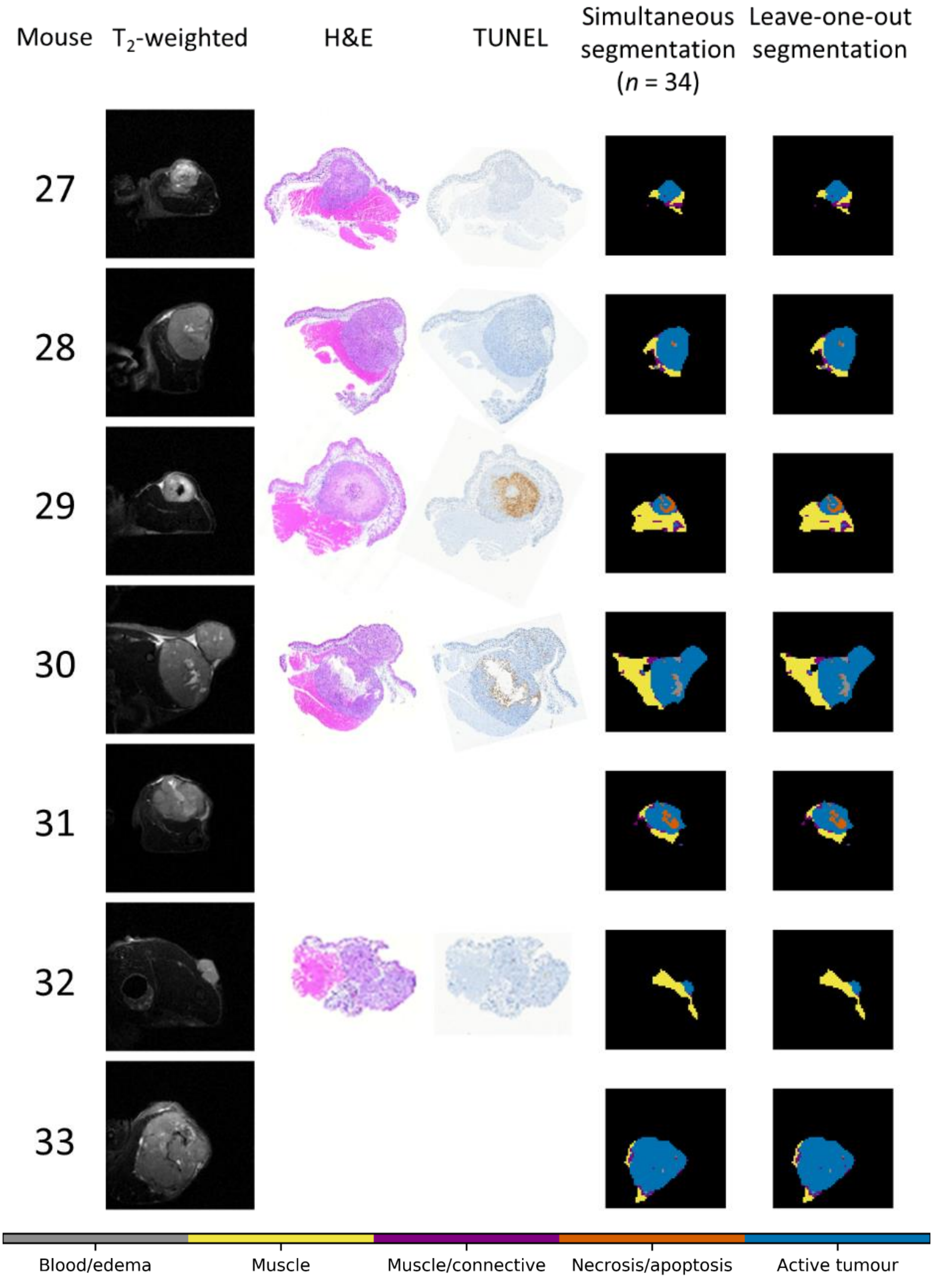
Mouse
T$_2$-weighted
H&E
TUNEL
Simultaneous segmentation (n = 34)
Leave-one-out segmentation
27
28
29
30
31
32
33
Blood/edema
Muscle
Muscle/connective
Necrosis/apoptosis
Active tumour

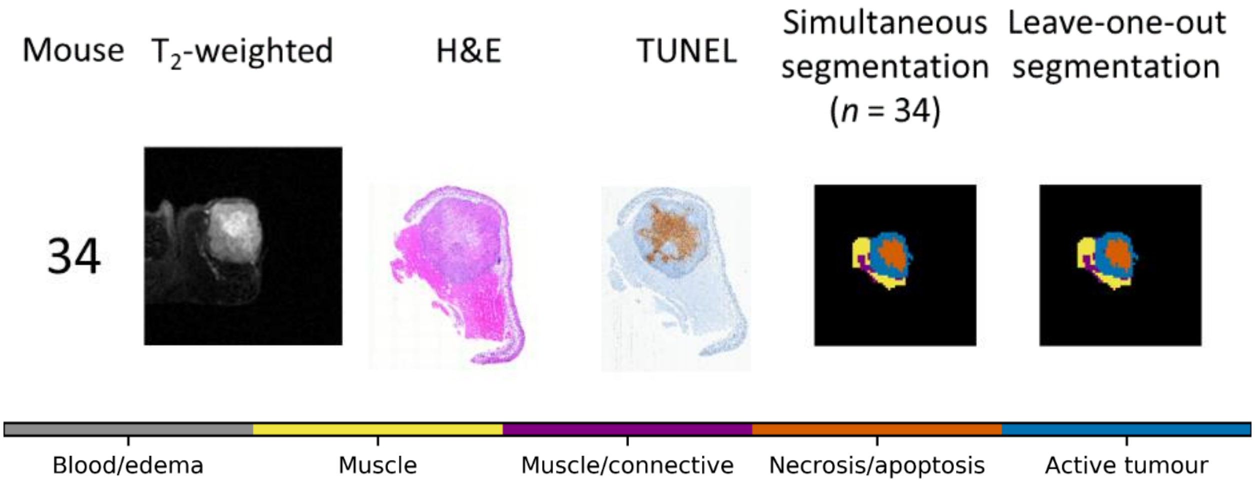

Figure S1. Comparison of whole-dataset and leave-one-out segmentation with anatomical images and histology for all mice. In all cases, the morphology and extent of the brown areas indicating necrosis/apoptosis in the TUNEL sections (third column) matched well with the segmentation masks calculated from whole-dataset (fourth column) and leave-one-out (fifth column) segmentations. The histology for mice #2, 5, 8, 11, 14, 19, 31, and 33 are omitted because they did not match the MR slice prescription or were not acquired in the days following MR imaging. The histology images for mice #17 and 18 only cover half their respective tumours due to processing errors and are so indicated by the boxes with a dashed line down the middle. It appears that the necrosis/apoptosis voxels in the leave-one-out segmentation mask for mouse #13 as erroneously clustered with the active tumour voxels. This is likely due to the proximity of active tumour and necrosis/apoptosis voxels in independent-component (IC) space, which confounded the Gaussian mixture model fitting. Preliminary analysis indicated that weighting the first IC values (i.e., stretching the voxels along IC_1) by a factor of two, which effectively spaces out the voxels in IC space, is sufficient to correct the error in the leave-one-out segmentation mask for mouse #13. Weighting of the IC values to increase robustness is left for future work. Note that the segmentation masks are cropped to include only voxels where the B_0 field offset is less than 0.5 ppm (i.e., where shimming was successfully applied).

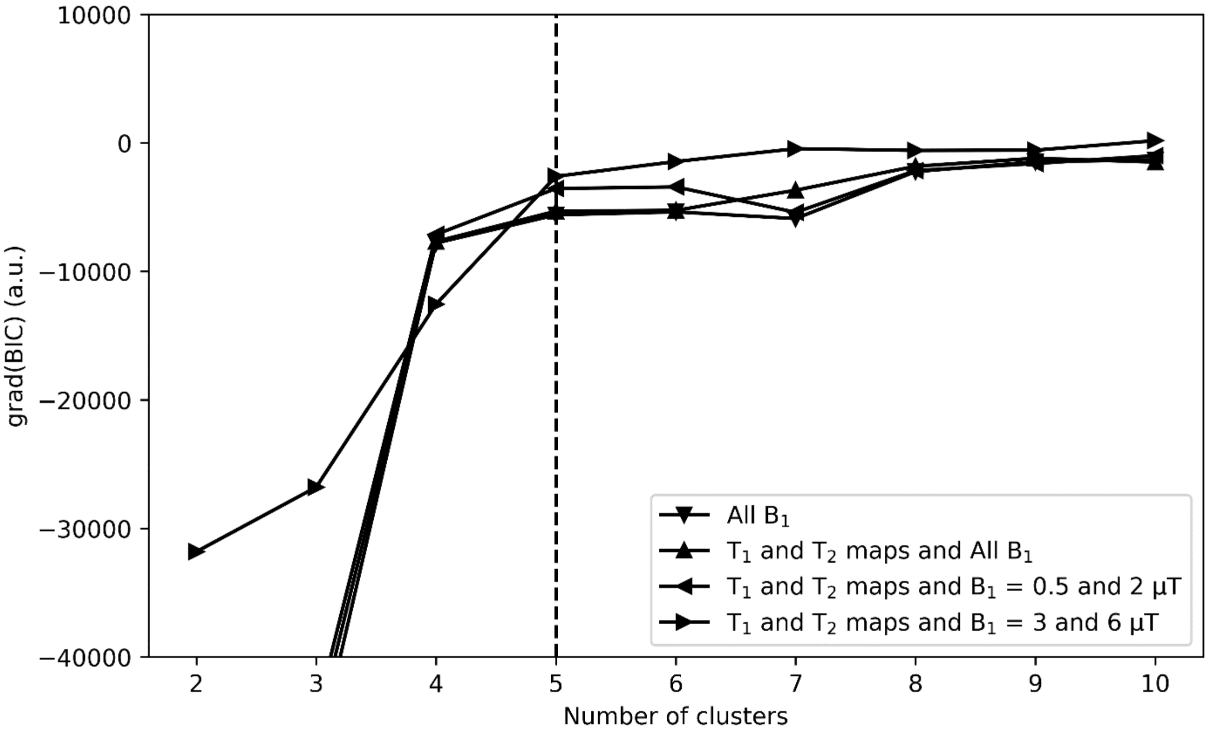

Figure S2. Determination of the number of clusters supported by the data. This is a plot of the gradient of the Bayesian information criterion (BIC) with respect to the number of clusters using four different sets of images as input to a three-component independent component analysis transform. The gradient of the BIC with respect to the number of clusters was calculated to give a function that sharply approaches zero until the optimal number of clusters and remains relatively constant afterwards. The dashed line at five clusters indicates the ideal number of clusters.

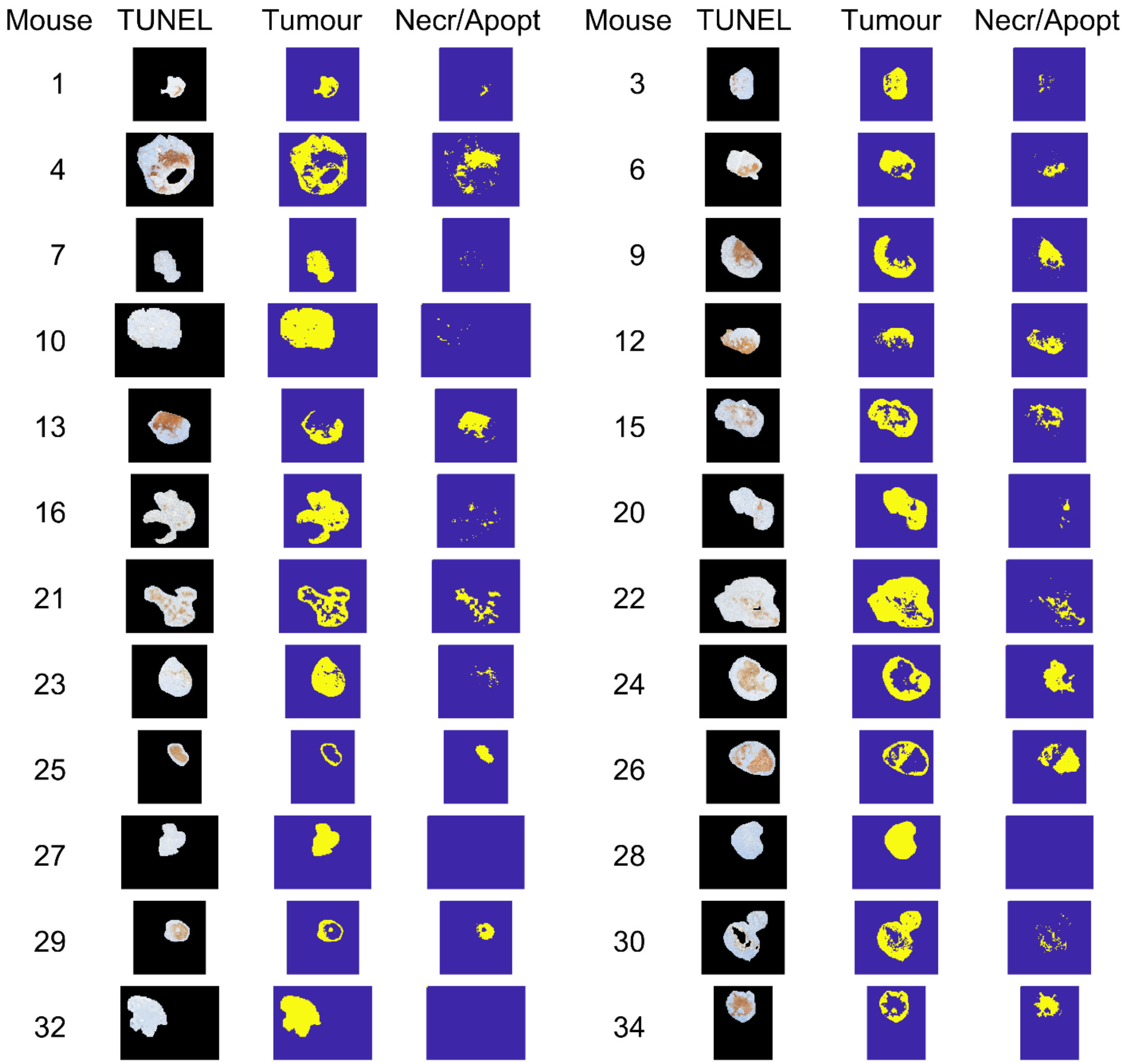

Figure S3. Segmentation of TUNEL histopathology sections into tumour and necrosis regions based on thresholding the blue channel of the RGB image after manual removal of muscle and skin voxels.

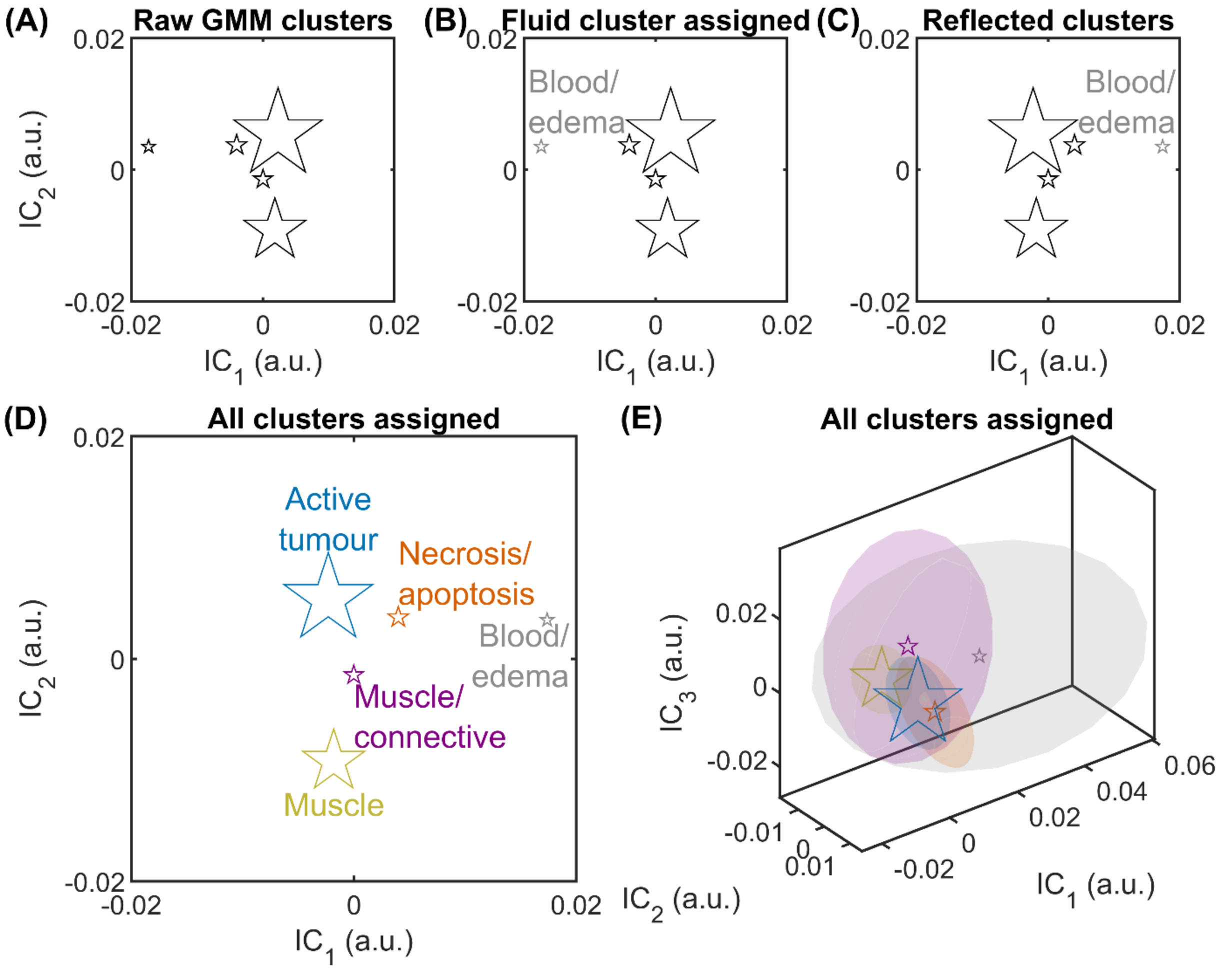

Figure S4. The cluster label assignment algorithm. (A) Raw Gaussian mixture model (GMM) cluster centroids (stars) estimated using the optimized protocol and data from all 34 mice. Although the clusters are in 3D independent component (IC) space due to the use of three ICs, a 2D projection is shown for clarity. (B) The cluster with the largest absolute value of IC_1 is labelled blood/edema (grey). (C) The clusters are reflected about the $IC_1 = 0$, $IC_2 = 0$, and $IC_3 = 0$ planes, as required, such that the blood/edema cluster is in the first octant for consistency, since independent component analysis does not identify the sign of the source signals. Here, only a reflection about the $IC_1 = 0$ plane was required. Then, the remaining clusters are assigned to muscle, muscle/connective, necrosis/apoptosis, and active tumour in order of increasing IC_2, shown in (D) 2D projection and (E) 3D. The variances of the Gaussians are only shown in E for clarity.

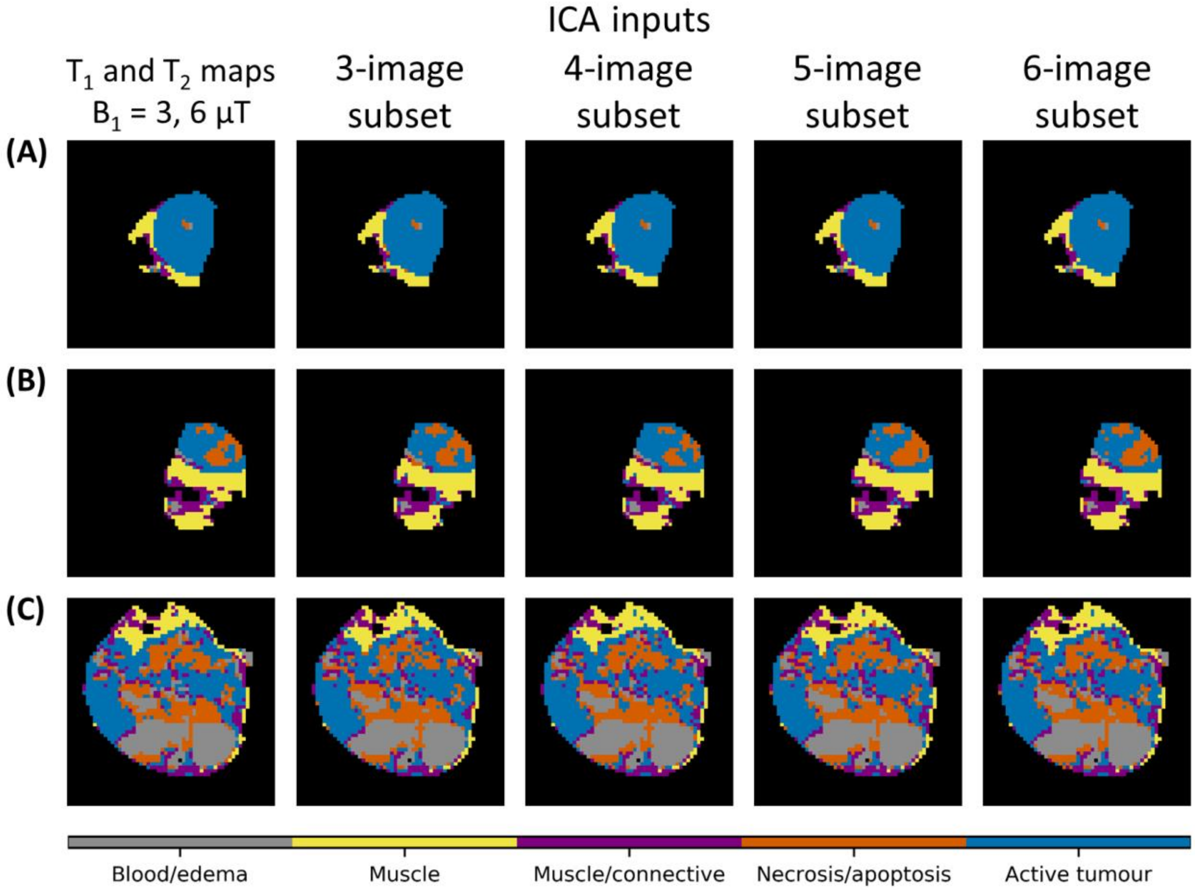

Figure S5. Comparison of segmentation using the three- to five-image subsets from Table 1 for the representative cases from Fig. 5. All the masks calculated using image subsets (second to fifth columns) have an excellent match to the masks calculated using all images (first column). All the masks were generated by segmenting the whole dataset (n = 34) simultaneously, i.e., leave-one-out segmentation was not used here.

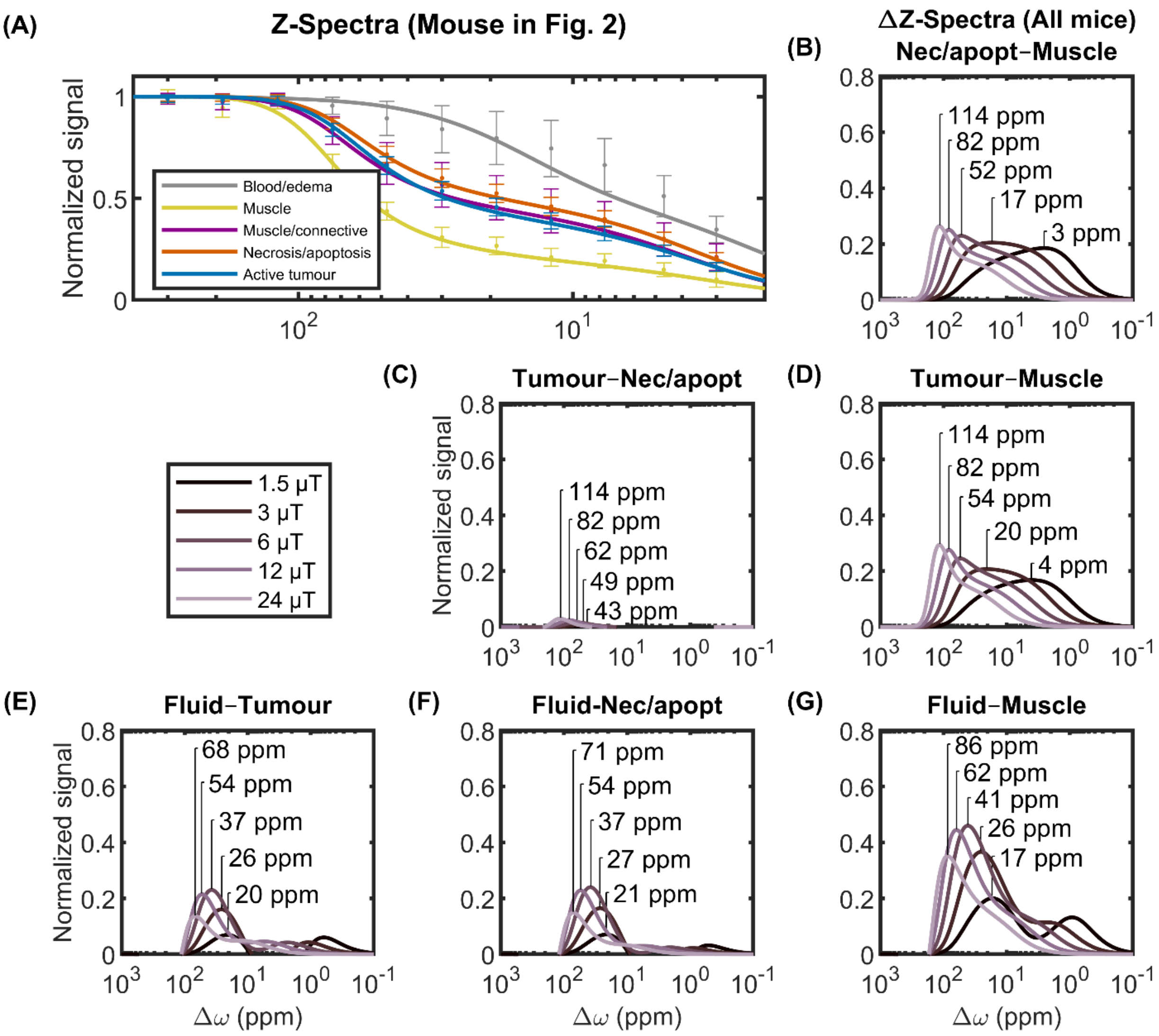

Figure S6. Representative quantitative MT model fit and Z-spectrum differences between clusters as a function of saturation amplitude. (A) Measured (points) and fitted (lines) Z-spectra with B_1 = 6 μT for the mouse in Fig. 2. Extrapolated Z-spectra from the mean estimated two-pool MT model parameters from fitting of all T_1 maps and Z-spectra acquired with B_1 = 0.1, 3, and 6 μT of (B) muscle voxels subtracted from those of necrosis/apoptosis (nec/apopt); (C) necrosis/apoptosis and (D) muscle from those of active tumour; and (E) active tumour, (F) necrosis/apoptosis, and (G) muscle from those of blood/edema (fluid). The frequency offsets Δω of the peaks are labelled. Z-spectrum differences with the muscle/connective voxels are not shown because they are similar to those calculated with active tumour voxels.

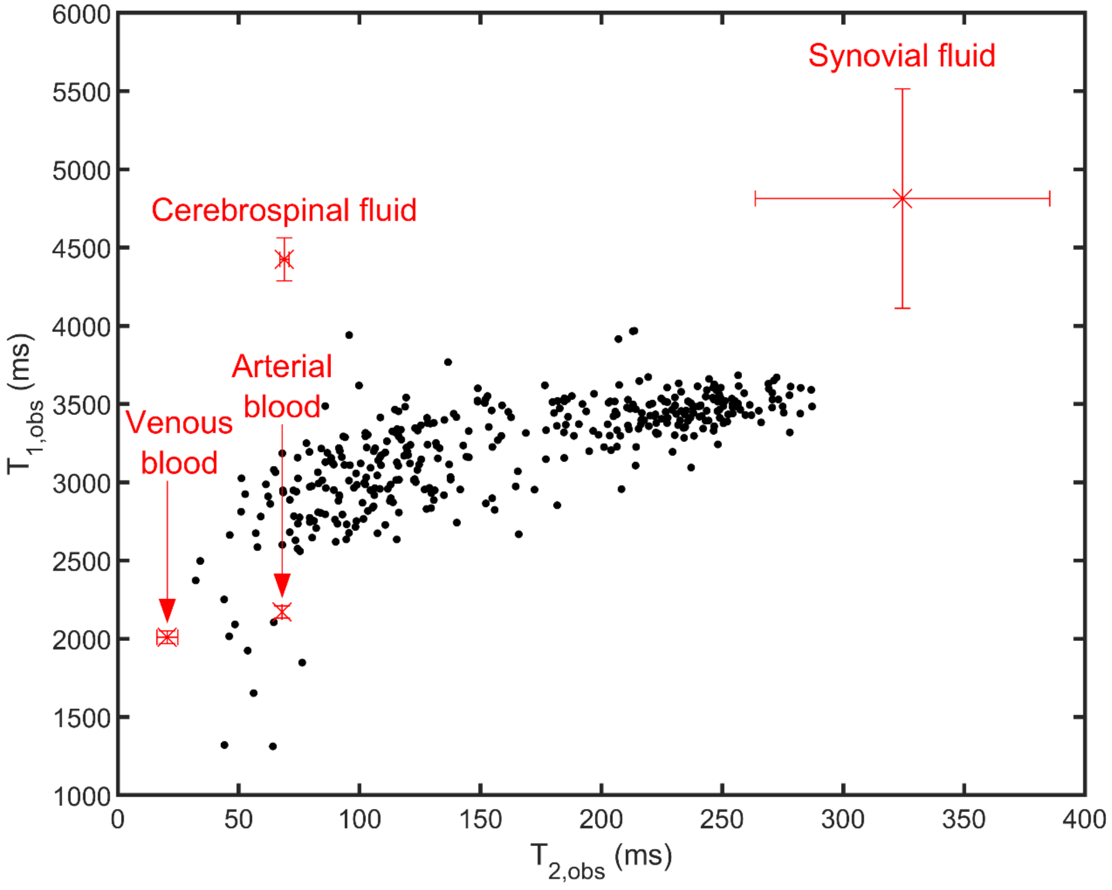

Figure S7. Scatter plot of the blood/edema voxels in the mouse in Fig. 2 as a function of their observed T_1 and T_2. Literature values for venous and arterial blood[1–3], cerebrospinal fluid[4–5], and synovial fluid[6] at 7 T (crosses) are given for reference. A minority of voxels appear to be blood, but there are an insufficient number of them to train the model.

REFERENCES

1. Zhang, X. *et al. Magn. Reson. Med.* **70**, 1082–1086 (2013).
2. Grgac, K. *et al. Magn. Reson. Med.* **70**, 1153–1159 (2013).
3. Krishnamurthy, L. C. *et al. Magn. Reson. Med.* **71**, 2035–2042 (2014).
4. Rooney, W. D. *et al. Magn. Reson. Med.* **57**, 308–318 (2007).
5. Liachenko, S. & Ramu, J. *J. Magn. Reson. Imaging* **45**, 700–709 (2017).
6. Jordan, C. D. *et al. Eur. J. Radiol.* **82**, 734–739 (2013).
